# Attenuation of epigenetic regulator SMARCA4 and ERK‐ETS signaling suppresses aging‐related dopaminergic degeneration

**DOI:** 10.1111/acel.13210

**Published:** 2020-08-04

**Authors:** Ling Sun, Jie Zhang, Wenfeng Chen, Yun Chen, Xiaohui Zhang, Mingjuan Yang, Min Xiao, Fujun Ma, Yizhou Yao, Meina Ye, Zhenkun Zhang, Kai Chen, Fei Chen, Yujun Ren, Shiwei Ni, Xi Zhang, Zhangming Yan, Zhi‐Rong Sun, Hai‐Meng Zhou, Hongqin Yang, Shusen Xie, M. Emdadul Haque, Kun Huang, Yufeng Yang

**Affiliations:** ^1^ Institute of Life Sciences Fuzhou University Fuzhou Fujian China; ^2^ Department of Medical and Molecular Genetics School of Medicine Indiana University Indianapolis IN USA; ^3^ MOE Key Lab of Bioinformatics School of Life Sciences Tsinghua University Beijing China; ^4^ Zhejiang Provincial Key Laboratory of Applied Enzymology Yangtze Delta Region Institute of Tsinghua University Jiaxing China; ^5^ Key Laboratory of Optoelectronic Science and Technology for Medicine Ministry of Education Fujian Normal University Fuzhou China; ^6^ Department of Biochemistry College of Medicine and Health Sciences United Arab Emirates University Al‐Ain United Arab Emirates; ^7^ Department of Hematology and Oncology School of Medicine Indiana University Indianapolis IN USA

**Keywords:** aging, *Drosophila*, MAPK‐ERK‐ETS signaling, neurodegeneration, Parkinson's disease, SMARCA4/Brahma

## Abstract

How complex interactions of genetic, environmental factors and aging jointly contribute to dopaminergic degeneration in Parkinson's disease (PD) is largely unclear. Here, we applied frequent gene co‐expression analysis on human patient *substantia nigra*‐specific microarray datasets to identify potential novel disease‐related genes. In vivo* Drosophila* studies validated two of 32 candidate genes, a chromatin‐remodeling factor SMARCA4 and a biliverdin reductase BLVRA. Inhibition of SMARCA4 was able to prevent aging‐dependent dopaminergic degeneration not only caused by overexpression of BLVRA but also in four most common *Drosophila* PD models. Furthermore, down‐regulation of SMARCA4 specifically in the dopaminergic neurons prevented shortening of life span caused by α‐synuclein and LRRK2. Mechanistically, aberrant SMARCA4 and BLVRA converged on elevated ERK‐ETS activity, attenuation of which by either genetic or pharmacological manipulation effectively suppressed dopaminergic degeneration in *Drosophila* in vivo. Down‐regulation of SMARCA4 or drug inhibition of MEK/ERK also mitigated mitochondrial defects in *PINK1* (a PD‐associated gene)‐deficient human cells. Our findings underscore the important role of epigenetic regulators and implicate a common signaling axis for therapeutic intervention in normal aging and a broad range of age‐related disorders including PD.

## Graphical abstract

Using bioinformatics analysis of large‐scale human transcriptomic data and Drosophila disease models, Sun et al. identified novel Parkinson's disease (PD) genes and revealed a potential common pathogenic signaling pathway consisting of the chromatin‐remodeling factor SMARCA4 and the ERK‐ETS signaling axis in normal aging and age‐related disorders such as PD. The cancer drug Trametinib, previously shown to have lifespan extending capacity, was found to have therapeutic potency in multiple PD disease models.

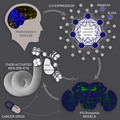

## 1 INTRODUCTION

Among age‐related diseases, Parkinson's disease (PD) is the most common neurodegenerative movement disorder, with an incidence rate above 1% among individuals over 65 years of age (Nalls et al., 2014). The pathologic manifestations of PD include age‐dependent progressive dopaminergic (DA) neuronal deterioration in basal ganglia and *substantia nigra*, with reduction of dopamine release. Remarkable similarities at the molecular and cellular levels exist between PD and normal aging. Current treatments for PD are only symptomatic, ameliorating disease symptoms for a limited period of time, without retarding or halting disease progression.

Parkinson's disease is a complex disease with high heterogeneity. Etiologically, PD consists of early‐onset subtypes, which are primarily due to high penetrance mutations and familial inheritance, and late‐onset subtypes, which occur more sporadically and are believed to result from complex interactions between genetic, environmental factors superimposed on the physiological decline of neuronal functions with age. Emerging evidence affirms the central role of genetic susceptibility in PD (Lill et al., 2012; Nalls et al., 2014). Although a comprehensive genetic architecture corresponding to distinct PD subtypes remains poorly understood, the same set of susceptibility genes may predispose people to both familial and sporadic PD. A number of causal genetic risk factors have been linked to PD onset, including mutations in *SNCA* (α‐synuclein), *LRRK2* (leucine‐rich repeat kinase 2), *VPS35* (the vacuolar sorting protein 35 gene), *EIF4G1* (eukaryotic translation initiation factor 4‐gamma) and *DNAJC13* [DnaJ heat shock protein family (Hsp40) member C13] genes with autosomal dominant inheritance mode, and *PARK2* (parkin), *PINK*1 (PTEN induced putative kinase 1), *PARK7* (Parkinsonism associated deglycase, *DJ1*), *HTRA*2 (high temperature requirement A2), *DNAJC*6 [DnaJ heat shock protein family (Hsp40) member C6], *FBXO7* (F‐box domain‐containing protein), *PLA2G6* (phospholipase A2 group VI), *SYNJ1* (synaptojanin 1), *ATP6AP2* (ATPase H+ transporting accessory protein 2) and *ATP13A2* (ATPase type 13A2) with recessive inheritance mode (Lin & Farrer, 2014).

Typically, these known PD genes participate in diverse cellular processes; however, common themes in PD pathogenesis have been proposed, such as aberrant proteostasis and vesicle trafficking, mitochondrial dysfunction, altered epigenetic regulation, and inflammation (Coppedè, 2013). Notably, all these involved pathogenic agents are similar to those in normal aging. Therefore, the insights involved in PD pathogenesis may be critical for understanding and modifying aging, and vice versa. Nevertheless, the majority of PD heritable components remain elusive (Lill et al., 2012; Nalls et al., 2014). How to extract contributing factors from limited human brain specimens is the main challenge to solve this heterogeneous disease, because only postmortem human brain specimens can be available. More challengingly, most postulated novel genetic associations or risk factors await further validation.

Gene co‐expression analysis allows identifying genes with similar expression patterns across a set of samples, which has facilitated identifying genes involved in certain disease pathways, new gene functions, and potential biomarkers (Zhang et al., 2012). In this study, we used the known PD genes as “anchors” in order to identify new PD candidate genes that are highly co‐expressed with known PD genes with multiple human brain microarray datasets. Among the predicted 32 candidate genes, SWI/SNF‐related, matrix‐associated, actin‐dependent regulator of chromatin, subfamily A, member 4 (*SMARCA*4) and biliverdin reductase A (*BLVRA*) were further studied in vivo using *Drosophila melanogaster*, and their potential involvement in PD pathogenesis was confirmed. Furthermore, we revealed a potential common aging‐related pathogenic signaling pathway consisting of the chromatin‐remodeling factor SMARCA4 and the ERK‐ETS signaling axis, suggesting new therapeutic targets for PD and other aging‐related disorders, as three ERK‐ETS inhibitors were tested for their efficacy in multiple *Drosophila* PD models. Our work also illustrates the high efficiency of combining bioinformatics analysis of large‐scale human transcriptomic data and small‐scale genetic screening using model organisms to interrogate highly heterogeneous diseases including age‐related disorders.

## 2 RESULTS

### 2.1 Gene co‐expression network analysis identified 32 novel PD‐associated candidate genes

We chose seven of the most commonly known PD genes as anchor genes, namely *SCNA*,* LRRK2*,* PARKIN*,* DJ1*, *PINK1*,* ATP13A2*, and *HTR2A*. Eleven datasets from NCBI Gene Expression Omnibus (GEO) were used, which contained samples from human brain tissues, especially the *substantia nigra* region. Our workflow is illustrated in Figure 1a. A total of 32 genes were identified to have high Pearson's correlation coefficients (PCC) with at least three anchor genes in at least five datasets (Table 1 and Table S1). According to the gene ontology (GO) enrichment analysis, the identified candidate PD genes were highly enriched with the genes associated with age‐dependent metabolic reprogramming and neural disorders (Blalock et al., 2004).

**FIGURE 1:**
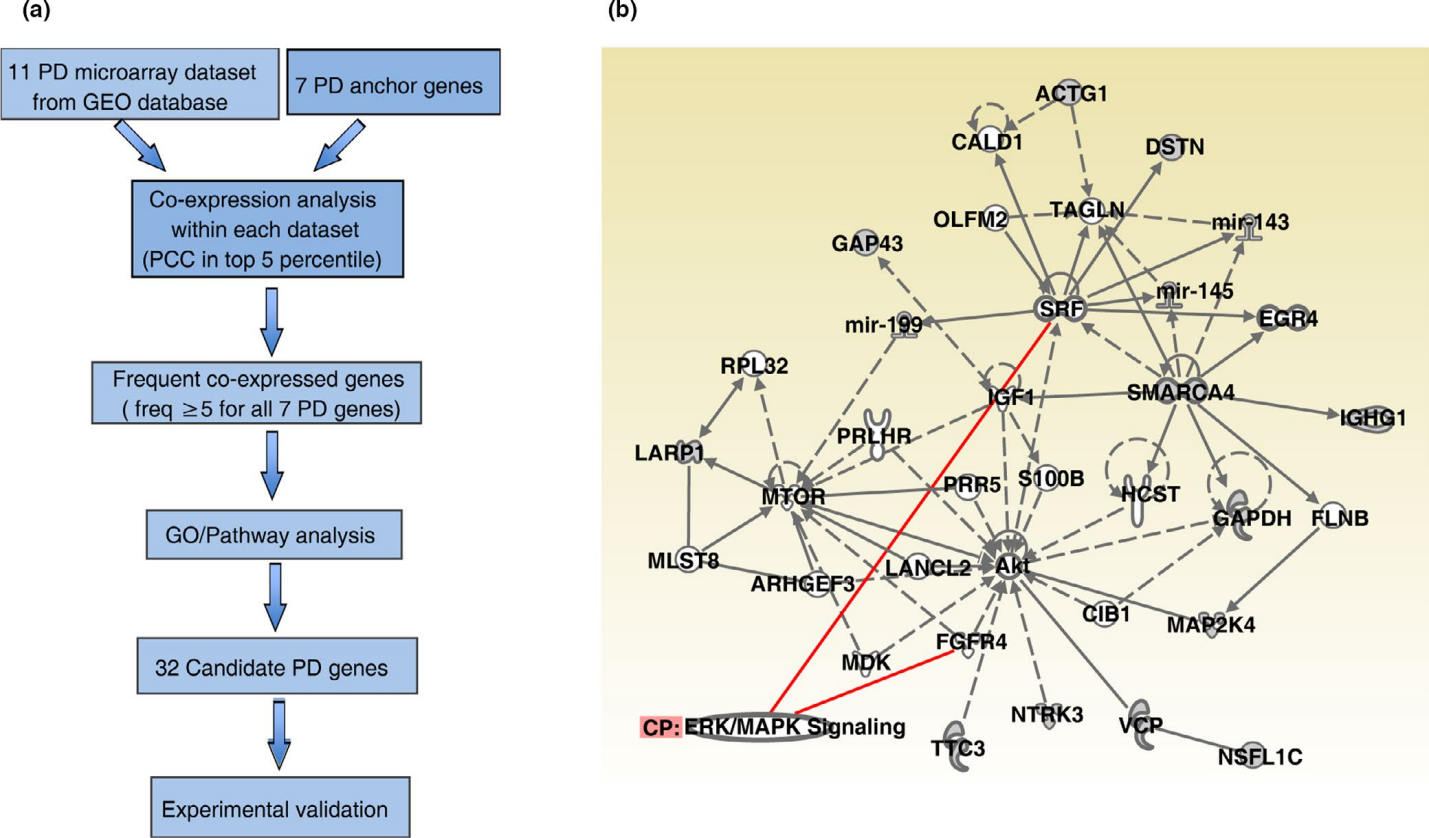
Bioinformatics analyses. (a) Overview of frequent gene co‐expression analysis in identifying novel Parkinson's disease‐associated genes. Eleven NCBI GEO DataSets were identified with the microarray datasets of the *substantia nigra* brain region of human PD patients. Seven genes were used as the anchor PD genes. See Section 4 for more details. (b) Ingenuity Pathway Analysis (IPA) revealed the connection of SMARCA4/Brm with MAPK kinase pathway. Red lines indicate genes known to interact with ERK signaling. Solid line: direct interaction; dash line: indirect interaction; arrows indicate the direction of interaction between two molecules. Different shapes indicate the type of the molecules

**TABLE 1:** Genes with highest anchor gene count (*N*[•]values) in at least five datasets

Anchor gene count (*N*)	Gene symbols
**5**	***SMARCA4***
4	*UBE3A, SNRPN, SLC25A3, PRDX2, GNAS, ARF1, ACTG1*
3	*VCP*,* TTC3*,* STMN2*,* RNF187*,* OBSL1*,* OAZ1*,* NTRK3*,* NSFL1C*,* MAP2 K4*,* LARP1*,* KLC1*,* IGHG1*,* GAPDH*,* GAP43*,* EPB41L1*,* DSTN*,* DKK3*,* CLTA*, ***BLVRA***,* ATP6V1C1*,* ATP5O*,* ATP5G3*,* ARF3*,* AAK1*

### 2.2 Inhibition of Brahma rescued progressive DA degeneration caused by overexpression of BVR in *Drosophila*

Among the 32 candidate genes, we chose SMARCA4 and BLVRA (biliverdin reductase A) for further studies based on the rationales below (Table S2). First, according to the Human Brain Transcriptome (HBT: https://hbatlas.org/pages/hbtd) and the Allen Brain Atlas (http://human.brain-map.org/), both SMARCA4 and BLVRA are expressed widely in the human brain, including the *substantia nigra*. Second, mutations of SMARCA4/Brm, a subunit of the SWI/SNF chromatin‐remodeling complex that regulates higher order chromatin structure and gene expression, have been linked to multiple neurological and psychiatric disorders including autism spectrum disorders and schizophrenia (De Rubeis et al., 2014; Koga et al., 2009). Although expression of SMARCA4/Brm has been reported in both murine and human DA neurons by recent single‐cell RNA‐seq profiling (Hook et al., 2018; Sandor et al., 2017), there has been no functional reports of its role for DA neurons yet. Biliverdin reductases (BLVRs), together with hemeoxygenases (HOs), constitute the evolutionarily conserved enzymes in the heme metabolism, exert multiple physiological functions, and have been considered as a potential biomarker for AD and mild cognitive impairment (Barone, Di Domenico, Mancuso, & Butterfield, 2014). We then queried with gene symbol “SMARCA4” and “BLVRA” in PD gene database PDGene (http://www.pdgene.org), which incorporates all available SNP data pertaining to the discovery phase of the GWAS meta‐analysis (Nalls et al., 2014). We found that both SMARCA4 (Table S3) and BLVRA (Table S4) harbor SNPs with meta‐analysis *p* value between 1E‐4 and 0.05, which can be regarded as the potential PD risk SNPs albeit not in the top 10,000 most significant GWAS results. Furthermore, both SMARCA4 and BLVRA are highly conserved among vertebrate and invertebrate species (Brahma or Brm for *Drosophila* SMARCA4 homologue and dBVR (CG9471) for *Drosophila* BLVRA homologue).

**FIGURE 2:**
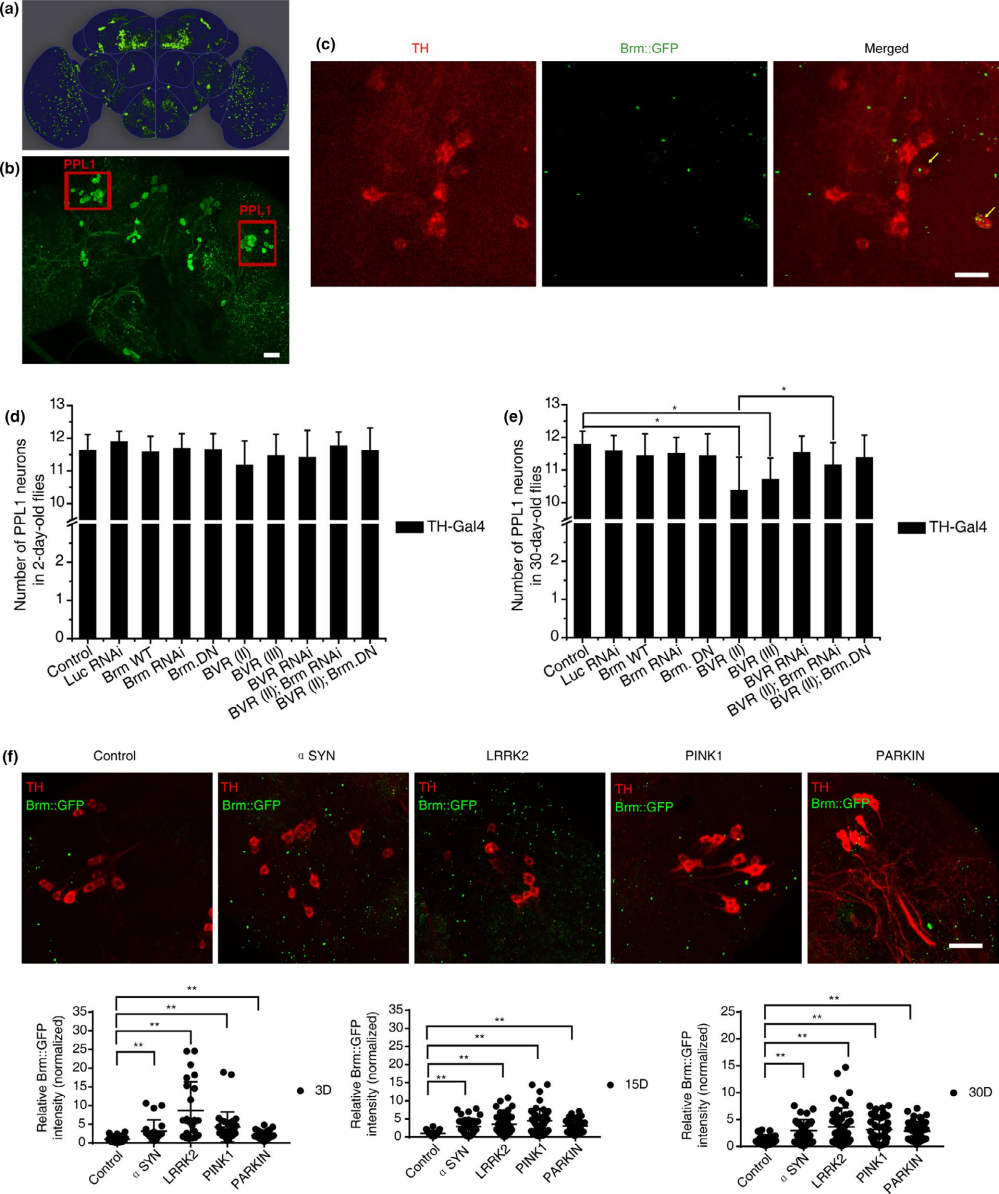
Inhibition of Brahma rescued dopaminergic (DA) degeneration caused by overexpression of BVR in *Drosophila*. (a) Schematic diagram of representative DA neuronal clusters in adult *Drosophila* brain. (b) Representative images show the posterior DA clusters in the fly brain. The intact left or right PPL cluster contains ~12 DA neurons at average in healthy adult flies. The DA neurons were labeled with green fluorescent protein (GFP). Scale bars, 25 μm. (c) Expression of Brm in the *Drosophila* DA neurons. A fusion Brm::GFP transgene was used to report the endogenous protein expression level of Brm. The Brm protein (green) was present in the *Drosophila* PPL1 DA neurons (anti‐TH, red) (pointed with yellow arrows) but not limited in DA neurons. Scale bars, 10 μm. (d, e) Scoring PPL1 DA neurons in 2‐day‐old (d) and 30‐day‐old (e) flies subjected to Brm‐ or BVR‐related genetic manipulations. Genetic manipulations included TH‐Gal4 driving overexpression of wide type Brm (Brm wt), a dominant‐negative allele of Brm (Brm^DN^), dBVR OE (Bvr II and Bvr III), Bvr II + Brm^DN^ and induction of Brm RNAi, BVR RNAi or Bvr II +Brm RNAi with w‐ (TH‐Gal4/+) and Luc RNAi (TH‐Gal4/UAS‐Luc RNAi) flies as the control. The genotypes of experimental flies are provided in the Appendix S2. Overexpression of dBVR resulted in DA neuronal loss in the aged fly brains when compared with controls (*Bvr* II, 10.37 ± 1.03; *Bvr* III, 10.70 ± 0.67; and control, 11.77 ± 0.42). n > 20 for each data point. (f) Progressive elevation of Brm protein levels (GFP signal) in the brains of four PD model flies in comparison to the control. Representative whole‐mount fluorescence images of fly brains are provided, with quantifications of the Brm protein level in young (3‐day‐old), middle‐age (15‐day‐old), and aged (30‐day‐old) flies shown below (*n* > 5). *indicates Mann–Whitney *p* < 0.01. Scale bar, 10 μm

To study the in vivo roles of candidate genes in PD pathogenesis, we took advantage of the *Drosophila melanogaster* model organism. The age‐dependent progressive DA neuronal loss in the lateral protocerebral posterior 1 (PPL1) cluster was used as the neurodegenerative index (Figure 2a–b). We then used an available Brm::GFP reporter fly strain to examine whether Brahma is expressed in fly DA neurons (Venken et al., 2011). Brm::GFP was first verified by its nuclear localization in *Drosophila* larval tissues (Figure S1a–c). Whole‐mount immunostaining then confirmed the expression of Brm in the fly DA neurons (Figure 2c), consistent with a previous report (Abruzzi et al., 2017).

*Drosophila* genetic manipulations were then carried out to dissect the roles of Brm and dBVR. When Brm RNAi‐mediated down‐regulation (Herr et al., 2010) or ectopic supply of a dominant‐negative allele of Brm (Brm^DN^) was induced specifically in DA neurons, no change in the number of PPL1 DA neurons was observed, neither was the overexpression of wild type Brm (Figure 2d,e). However, pan‐neuronal (*elav*‐Gal4 driver) overexpression or down‐regulation of Brm led to early lethality. Meanwhile, we generated two UAS‐dBVR transgenic fly strains, Bvr II and Bvr III, which enabled overexpression of dBVR (Figure S2a,b). Pan‐neuronal or DA neuron‐specific overexpression of dBVR resulted in PPL1 DA neuronal loss in the aged fly brains as compared with controls, while an available dBVR RNAi line did not show any effects (Figure 2d, e, Figure S2c,d). Interestingly, no global defects were detected in the fly brains with pan‐neuronal overexpression of dBVR, implicating that the DA neurons were specifically vulnerable to elevated dBVR level. Remarkably, ectopic supply of Brm^DN^ suppressed the progressive DA loss caused by dBVR overexpression (Figure 2d,e). The rescuing effect was not owing to titration of UAS‐mediated overexpression (Figure S3).

### 2.3 Brahma was upregulated in multiple *Drosophila* PD models

We then examined how Brm and dBVR genetically interact with known PD genes. Four previously reported *Drosophila* PD models were successfully reproduced in our laboratory (Clark et al., 2006; Feany & Bender, 2000; Greene et al., 2003; Imai et al., 2008; Park et al., 2006; Yang et al., 2006). Given that the homozygous Parkin null alleles were found to be unhealthy in our laboratory, we used Parkin RNA interference (RNAi) flies instead. These four *Drosophila* PD models were abbreviated as αSyn (A30P), LRRK2 (I1915T), Parkin, Pink1 (Pink1 mut or Pink1 RNAi) PD models, respectively (see Section 4 & fly genotypes listed in the Appendix S2). Consistent with previous findings, age‐dependent progressive degeneration was mild but statistically significant, as evidenced by the decreased number of PPL1 DA neurons in 30‐day‐old flies compared with age‐matched controls. In contrast, young 2‐day‐old PD flies displayed no DA neuronal loss (Figure S4).

By labeling DA neurons with red fluorescent protein (RFP) in parallel, we found that Brm::GFP level exhibited an age‐dependent progressive elevation in the PD fly brains compared with controls (Figure 2f). We next addressed how Brm is upregulated in the degenerative dopaminergic neurons. One possibility is there could be a link between Brm activity and oxidative stress, which has been widely believed to be a common pathogenic factor in PD. To this end, we monitored the oxidative stress indicated by the ROS fluorescent dye (DCF‐DA) (Figure S5) or the reduction–oxidation‐sensitive GFP (roGFP) (Figures S6 and S7). We also evaluated the level of anti‐oxidant response in the brains of PD model flies using GstD‐GFP as a reporter (Sykiotis & Bohmann, 2008) (Figure S8). To our surprise, unlike Brm which was pronouncedly elevated at 15d AE during the disease progression in all four PD models, no substantial increase of brain oxidative stress reporting signal was detected until 15‐20d AE in the brains of PD flies except for the αSyn detected by roGFP (Figures S6 and S7). Therefore, it implicates that Brm up‐regulation in PD flies might not be largely due to the change of DA neuronal oxidative stress.

### 2.4 Down‐regulation of Brahma specifically in the DA neurons restored life span in the *Drosophila* PD models

**FIGURE 3:**
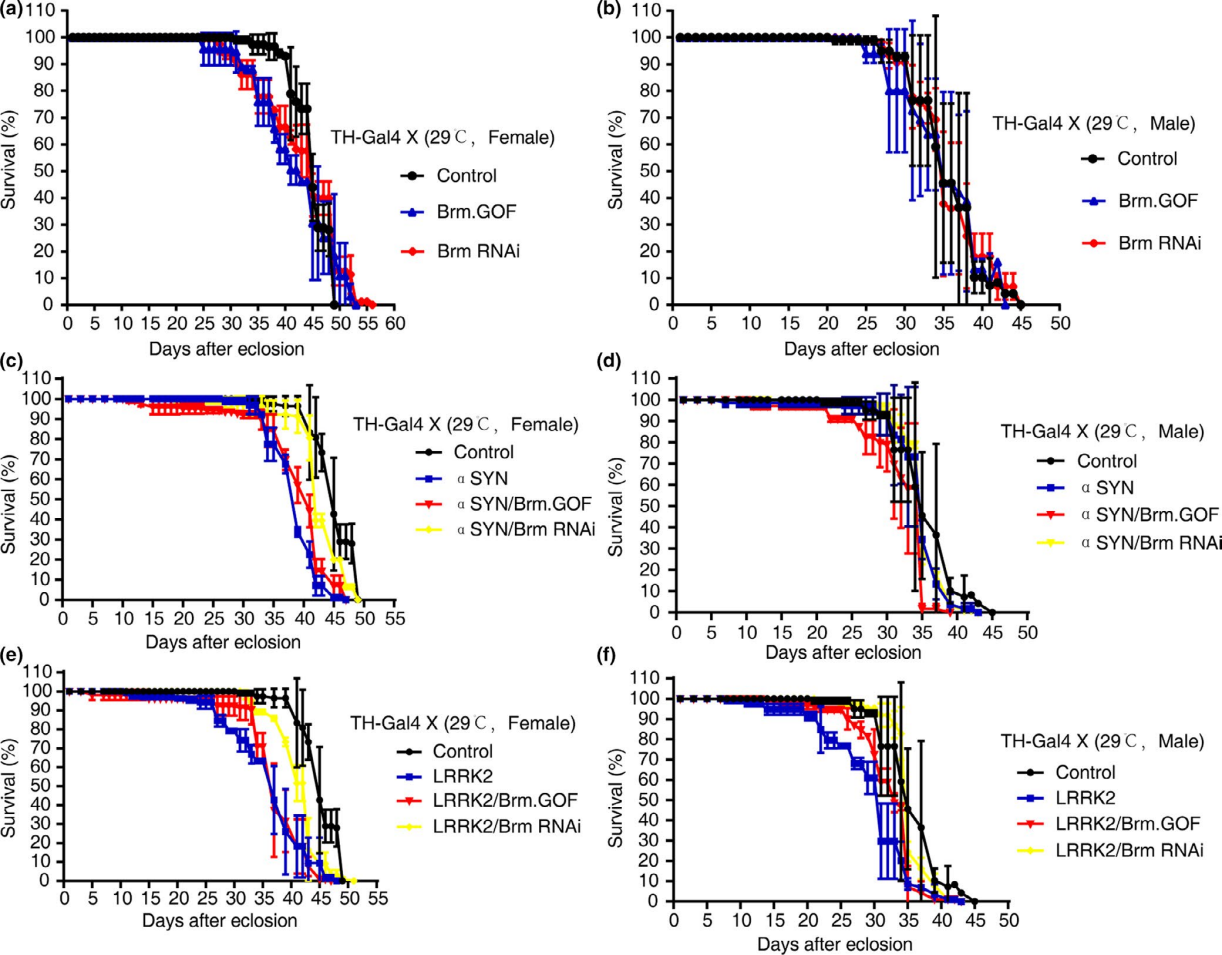
Down‐regulation of Brahma in the dopaminergic neurons prevented shortening of life span in *Drosophila* PD models. (a, b) Perturbation of Brm activity did not significantly affect the life span of *Drosophila* in comparison to that of control flies (TH‐Gal4/+). Overexpression of a dsRNA construct or a constitutively activated allele of Brm was induced specifically in the DA neurons, respectively. The life span of both female and male flies was evaluated separately. (c, d) Down‐regulation of Brm partially restored the median life span in the female flies in α‐synuclein‐mediated PD model, while over‐activation of Brm did not have an aggravating effect. Note that the life span of male flies with α‐synuclein overexpression was relatively normal. (e, f) Down‐regulation of Brm suppressed the shortening of the median life span of both female and male flies in LRRK2‐mediated PD model; in contrast, over‐activation of Brm did not have discernible impacts

When Brm was downregulated or upregulated specifically in the *Drosophila* DA neurons, only slight perturbances in the life span were observed (Figure 3a,b, Table S4). In line with previous reports (Imai et al., 2008; Todd & Staveley, 2008), overexpression of αSyn (A30P) or LRRK2 (I1915T) led to significantly shortened median life span of *Drosophila* without affecting the maximal life span (Figure 3c–f, Table S4). Remarkably, when Brm was downregulated specifically in the DA neurons of PD model flies, the shortening of the median life span of both female and male *Drosophila* was prevented (Figure 3c–f, Table S4), while overactivation of Brm did not exhibit discernible effect (Figure 3c–f, Table S4), implicating that proper Brm levels might be crucial in healthy aging.

### 2.5 Genetic manipulations of Brm or BVR modulated DA degeneration in multiple *Drosophila* PD models

When Brm RNAi was introduced into the four different PD model flies, significant suppression of PPL1 DA neuronal loss was detected in the aged flies (Figure 4a–d). As a control, an introduction of irrelevant Luc RNAi did not exhibit such inhibitory effects (Figure 4a–d). Accordingly, overexpression of Brm^DN^ fully prevented the progressive PPL1 DA neuron degeneration in all four PD fly models, although overexpression of Brm in the PD context did not induce further neuronal loss (Figure 4a–d and Figure 2e). The observed rescuing effects did not occur during the developmental stage, since the inhibition of Brm alone did not increase the number of DA neurons more than normal in the young flies (Figure 2d and Figure S9a). Neither was the rescuing effect due to titration of UAS‐mediated overexpression (Figure S3).

**Figure 1:**
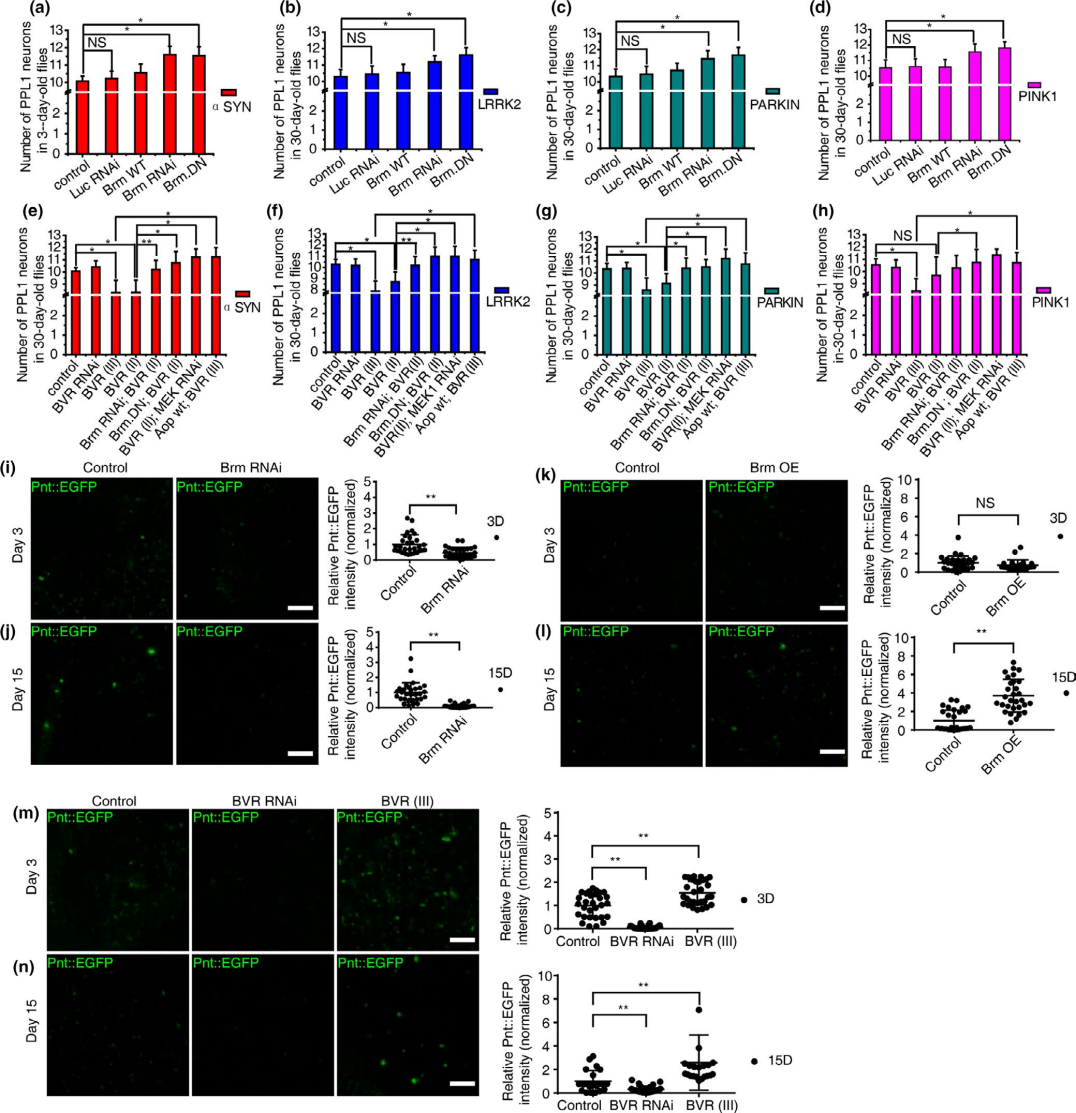
Intervening Brahma or BVR modulated dopaminergic (DA) degeneration in multiple established *Drosophila* PD models. (a–d) RNAi knockdown of Brm prevented DA neuronal loss in four different forms of parkinsonian DA degeneration: αSyn (a), LRRK2 (b), Parkin RNAi (c), and Pink1 mut (d) models. DA degeneration was revealed in the neuronal loss in the PPL1 DA neuron clusters (*n* > 20). Suppression of Brm by overexpression of the dominant‐negative of Brm (Brm^DN^) rescued DA neuronal loss in all four PD fly models (*n* > 20). (e–h) Overexpression of dBVR aggravated the progressive DA neuronal loss in aged four PD model flies (αSyn, 8.31 ± 1.01; Lrrk2, 7.89 ± 0.90; Pink1 mut, 8.42 ± 1.00; and Parkin RNAi, 8.58 ± 1.00), which could be suppressed by MEK RNAi or Aop^wt^ overexpression in all four PD models; the same effects were also found with Brm^DN^ overexpression (*n* > 20). (i–n) Brm and dBVR positively correlated with the ERK‐ETS signaling level indicated by the fusion protein Pnt::EGFP, which reflects the endogenous protein expression level of Pnt. Red fluorescent protein (RFP) was used to label DA neurons. Representative whole‐mount fluorescence images of brains of different ages are shown, with the corresponding quantification of Pnt protein level displayed on the right‐side panels (*n* > 5). The genotypes of experimental flies are provided in the Appendix S2. *indicates Mann–Whitney *p* < 0.05, **indicates *p* < 0.01. NS, not significant. Scale bar, 10 μm

On the other hand, overexpression of dBVR aggravated the progressive DA neuronal loss in aged four PD model flies, while dBVR RNAi did not mitigate the degeneration (Figure 4e–h and Figure S9b). Importantly, the aggravation caused by dBVR overexpression could be fully rescued through the addition of Brm^DN^ in all four PD model flies and in three PD models (except the *PINK1* deficiency model) through Brm RNAi (Figure 4e–h). The rescuing effect was not owing to titration of UAS‐mediated overexpression (Figure S3). Collectively, these results demonstrated that inactivation of Brm protects DA neurons from age‐dependent degeneration in a variety of pathogenic genetic backgrounds.

### 2.6 Prolonged over‐activated MEK‐ERK‐ETS signaling in multiple PD fly models

We then investigated the possible mechanisms through which Brm and dBVR could affect DA degeneration. An Ingenuity Pathway Analysis (IPA) revealed the connection of SMARCA4/Brm with ERK signaling pathway (Figure 1b), as was supported by a previous study (Herr et al., 2010). On the other hand, hBVR has been previously suggested as an ERK activator in HEK293A cells (Lerner‐Marmarosh, Miralem, Gibbs, & Maines, 2008). Activator *Pointless* (Pnt) and repressor *Anterior open* (Aop) are two downstream antagonizing players of the MEK‐ERK signaling and both belong to E‐twenty six transcription factors (O'Neill, Rebay, Tjian, & Rubin, 1994). We used the reporter fly line of Pnt::EGFP (O'Neill et al., 1994) to monitor the MEK‐ERK activity in the fly brain and found that both Brm RNAi and dBVR RNAi resulted in reduced MEK‐ERK activity in fly DA neurons, while overexpressing either Brm or dBVR upregulated the signaling (Figure 4i–n). Conversely, DA neuron‐specific knockdown of *Drosophila* MEK (*Dsor1*) by RNAi (Slack et al., 2015) or overexpression of a wild type allele of the negative regulator of ERK pathway, Aop*^[wt]^*, suppressed the aggravation of progressive DA degeneration induced by dBVR overexpression in four PD fly models (Figure 4e–h); Aop*^[wt]^* overexpression also prevented DA degeneration caused by dBVR overexpression alone (Figure S10). These rescuing effects were not due to titration of UAS‐mediated overexpression (Figure S3).

**FIGURE 5:**
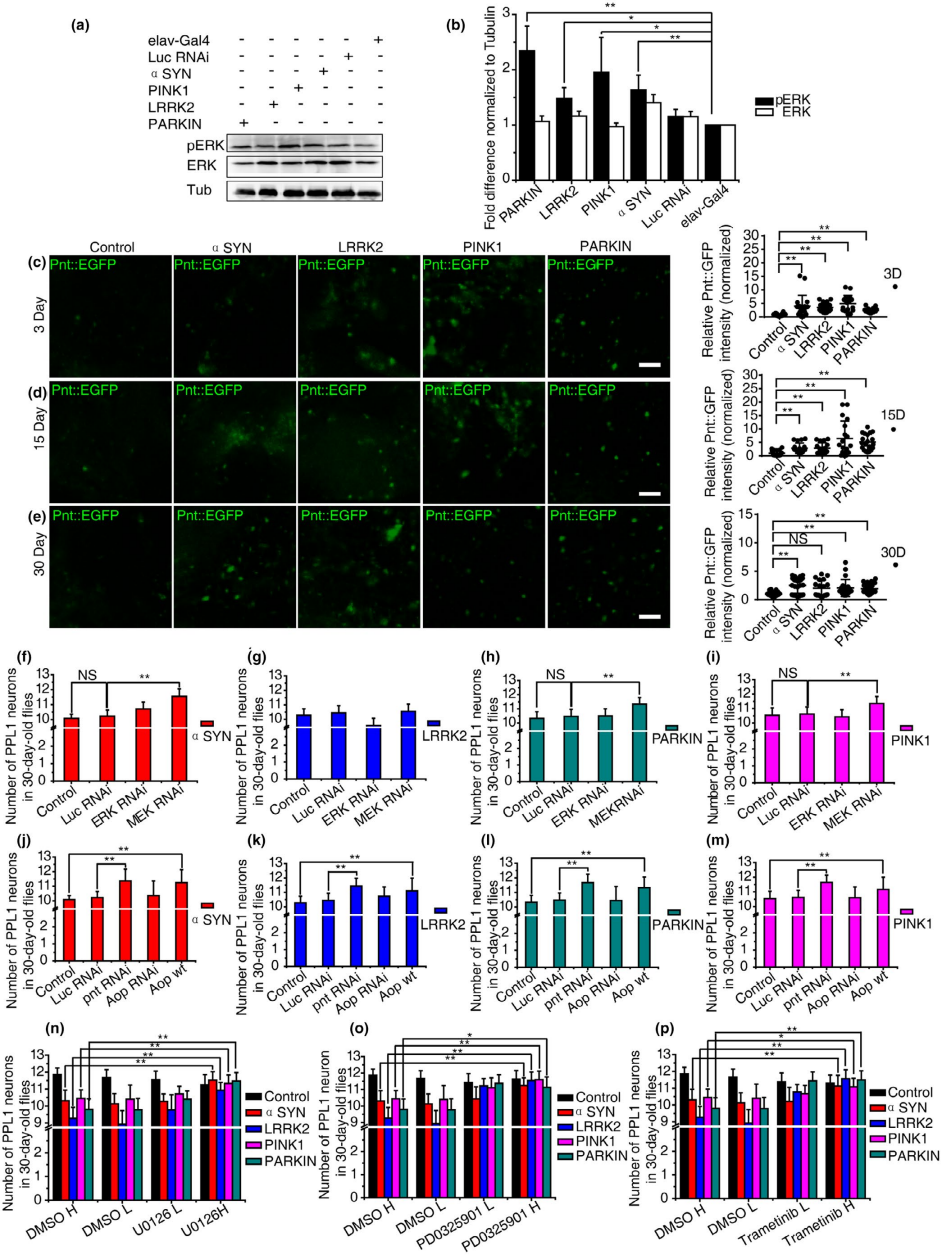
Attenuation of MEK‐ERK‐ETS signaling activity prevented dopaminergic (DA) degeneration in *Drosophila*. (a, b) The ERK phosphorylation level was significantly enhanced in all four PD model fly brains with *elav*‐Gal4 as the driving line. Luc RNAi was used as a control for Pink1RNAi and Parkin RNAi experiments. (c–e) Sustained elevated Pnt level in PD fly brains indicated prolonged over‐reactive MEK/ERK signaling. Pnt::EGFP and RFP were used and quantified as in Figure 4. Representative whole‐mount fluorescence images of brains of different ages are shown. (f–i) Knockdown of MEK prevented the deterioration of the PPL1 DA neurons in three PD fly models: αSyn, 11.55 ± 0.5 (f); Parkin RNAi, 11.33 ± 0.47 (h); Pink1 mut, 11.36 ± 0.48 (i); and control, 11.67 ± 0.22. (j–m) Knockdown of pnt prevented the deterioration of the PPL1 DA neurons in all four PD fly models: αSyn, 11.36 ± 0.81 (j); Lrrk2, 11.44 ± 0.53 (k); Parkin RNAi, 11.67 ± 0.52 (l); Pink1 mut, 11.65 ± 0.49 (m); and control, 11.42 ± 0.67. Overexpression of Aop^wt^ suppressed the degeneration of the PPL1 DA neurons in all four PD fly models: αSyn, 11.25 ± 0.89 (j); Lrrk2, 11.1 ± 0.88 (k); Parkin RNAi, 11.33 ± 0.72 (l); Pink1 mut, 11.17 ± 0.83 (m); and control, 11.53 ± 0.32. (n–p) Oral administration of three MEK inhibitors suppressed DA degeneration in *Drosophila*, respectively. Two concentrations of either U0126 (n) (low [L]: 1 μg/ml; high [H]: 10 μg/ml), PD0325901 (o) (L: 1 μg/ml; H: 10 μg/ml), or Trametinib (p) (L: 1.6 μM; H: 16 μM) were applied (*n* > 20). The genotypes of experimental flies are provided in the Appendix S2. *indicates Mann–Whitney *p* < 0.05, **indicates *p* < 0.01. NS, not significant. Scale bar, 10 μm

We then examined whether overactivation of ERK‐ETS was prevalent in those common PD fly models. Remarkably, we observed increased phosphorylated ERK (pERK) levels in all four PD model fly brains (Figure 5a,b), which were concordant with sustained up‐regulation of Pnt (Figure 5c–e).

### 2.7 Attenuation of MEK‐ERK‐ETS activation prevented DA degeneration in multiple *Drosophila* PD models

We next examined whether directly reducing MEK‐ERK‐ETS activation was sufficient to prevent those common forms of DA degeneration. MEK RNAi fully rescued PPL1 DA neuronal loss in three 30‐day‐old PD model flies (Figure 5f–i, Figure S11a,b), and the rescue was not owing to titration of UAS‐mediated overexpression (Figure S3). In contrast, *Drosophila* ERK (*rolled* or *rl*) RNAi (Slack et al., 2015) exerted no apparent rescuing effects, suggesting that it was the activated fraction of ERK (pERK), not the abundance of the ERK protein, that caused the neurotoxicity. In parallel, when MEK RNAi or ERK RNAi was induced alone specifically in DA neurons, no changes in the PPL1 DA neurons were observed when compared with age‐matched controls, suggesting that the rescuing effects of MEK RNAi were epistatic, but not due to simple addition (Figure S11a). Overexpression of constitutively active ERK led to early larval lethality before eclosion. We further found that the DA neuron‐specific overexpression of a constitutively active form of Pnt (*Pnt^[P1]^*) led to early lethality before eclosion too. In addition, DA neuron‐specific RNAi knockdown of Pnt (Slack et al., 2015) led to a rescue or mitigation. Remarkably, DA neuron‐specific overexpression of the negative regulator, *Aop^[wt]^*, completely blocked the aging‐related PPL1 DA neuronal loss in all four PD model flies (Figure 5j–m and Figure S12a,c), and the rescue was not owing to titration of UAS‐mediated overexpression (Figure S3). No further aggravation was observed upon DA‐specific RNAi (Slack et al., 2015) knockdown of *Aop* (Figure 5j–m, Figure S11a,c). Similar to Pnt, DA neuron‐specific overexpression of a constitutively active form of AOP (*AOP^[CA]^*) led to early lethality before eclosion, and overexpression of *Aop^[wt]^* or Aop RNAi alone resulted in mild DA neuronal loss (Figure S11a). Based on all the above evidences, we conclude that there might be a delicate range of the ERK‐ETS signaling strength that is beneficial for the maintenance of DA neurons, while deviation from that range such as prolonged over‐activation could be rather detrimental.

### 2.8 Pharmacological inhibition of ERK signaling suppresses DA neuronal loss in multiple *Drosophila* PD models

To examine the MEK/ERK signaling pathway as a drug target for intervening in DA degeneration, we started with the MEK1 inhibitor, U0126. The effective inhibitory dose was first determined in flies subjected to 7 days of drug feeding (Figure S12a,b). After continuous oral supplementation of U0126 (10 μg/ml) in adult flies for 30 days, DA neuronal loss in PPL1 clusters was blocked effectively in all four PD fly models (Figure 5n). Exposure to another MEK1 inhibitor, PD0325901 (10 μg/ml), also completely blocked PPL1 DA neuronal loss (Figure 5o). We further tested Trametinib, another potent and highly specific MEK1 inhibitor, an FDA‐approved drug for the treatment of melanoma (Yamaguchi, Kakefuda, Tajima, Sowa, & Sakai, 2011). With the optimal feeding concentration of 16 μM of Trametinib (Figure S12c,d) (Slack et al., 2015), PPL1 DA neurons were fully protected from degeneration (Figure 5p). No global brain or motor behavioral abnormalities were detected with all these drug treatments. Taken together, our data demonstrated that the MEK‐ERK pathway could be a valid drug target to revert DA degeneration and illustrated that oral administration could be a promising pharmacological intervention.

### 2.9 Knockdown of *SMARCA4* or drug inhibition of ERK activity ameliorated mitochondrial defects in *PINK1*‐deficient human cells

**FIGURE 6:**
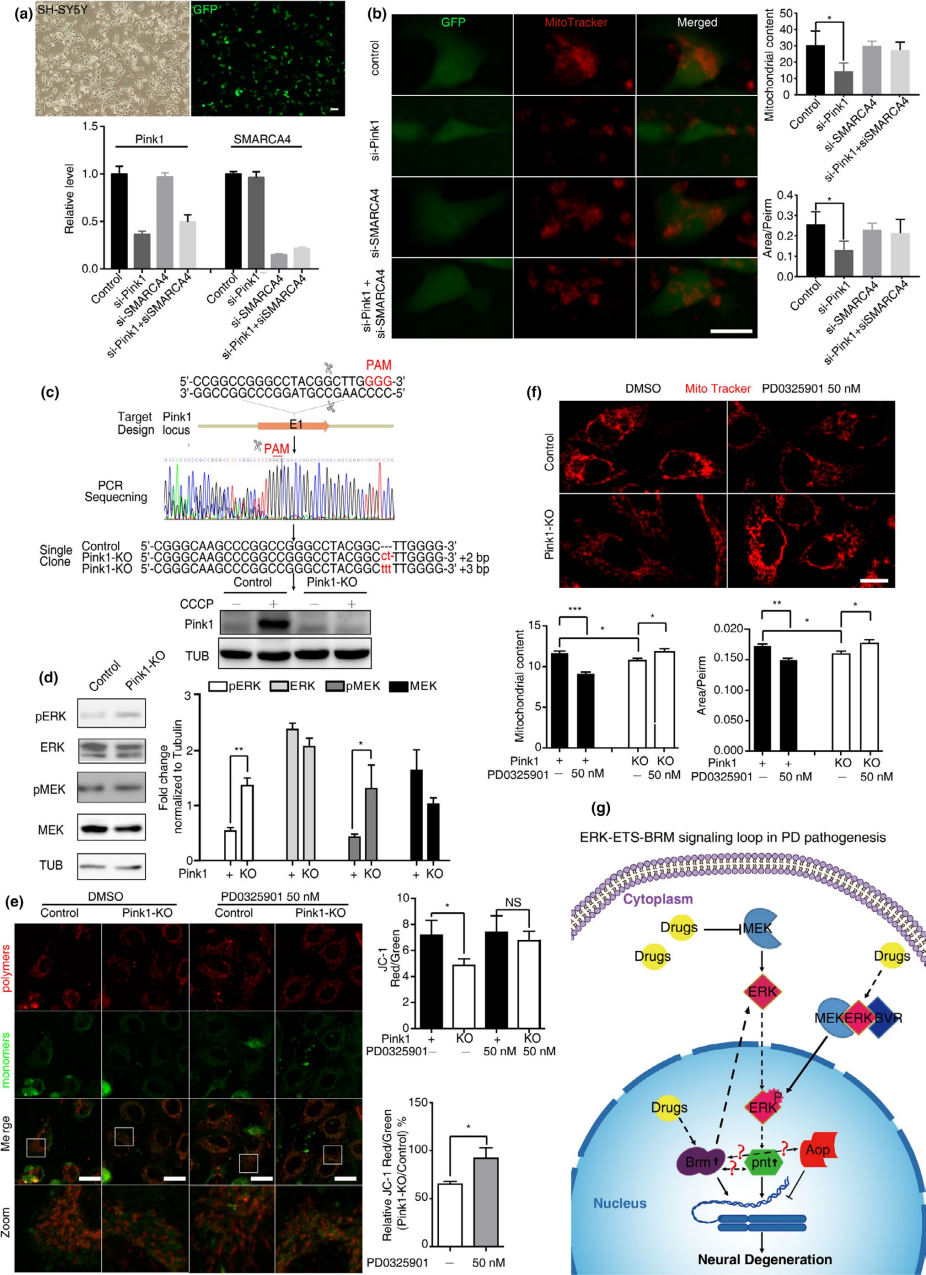
Knockdown of *SMARCA4* or drug inhibition of MEK‐ERK signaling ameliorated mitochondrial defects in the *PINK1 deficient* human cells. (a) Knockdown of *SMARCA4* and *PINK1* by RNAi in SH‐SY5Y cells. Cells were transfected with interference dsRNAs of *PINK1*, interference dsRNAs of *SMARCA4*, interference dsRNAs of *PINK1* and *SMARCA4*, respectively, interference dsRNAs of Luciferase were transfected as RNAi control. Relative expression of *PINK1* and *SMARCA4* were quantified, at least 3 repeats per column were calculated. Scale bars, 10 μm. (b) *SMARCA4* knock down significantly mitigated the aberrant mitochondrial content and interconnectivity caused by *PINK1* RNAi SH‐SY5Y cells. Mitochondria were stained with MitoTracker‐Red. The mean mitochondrial content and mitochondrial interconnectivity index (Area/Perim) are shown as mean ± *SD*. *: indicates Mann–Whitney *p* < 0.05, **: indicates *p* < 0.01. NS, not significant. Scale bars, 10 μm. (c) CRISPR/Cas9 to establish the *PINK1* ^−/−^ HeLa cell line. Schematic overview of the strategy used to generate the *pink1* knockout allele, Sanger sequencing was used to identify the targeted mutations, and Western blot analysis verified the loss of PINK1 protein. Control or *PINK1* KO HeLa cells, untreated (‐) or treated (+) with the protonophore m‐chlorophenylhydrazone (CCCP) were used; CCCP was used to prevent PINK1 protein from degradation (Narendra et al., 2010). (d) Over‐activated MEK/ERK signaling in *PINK1* ^−/−^ HeLa cells. (e) Inhibition of MEK‐ERK signaling by PD0325901 rescued mitochondrial membrane potential (MMP) defects in *PINK1* ^−/−^ HeLa cells. The mean ratio of the JC‐1 dye intensity in red channel to green channel is shown as mean ± *SD* to quantify MMP. Relative MMP between *PINK1* ^−/−^ and control is also shown. (f) Inhibition of MEK/ERK signaling improved mitochondrial content and mitochondrial interconnectivity in *PINK1* ^−/−^ HeLa cells. Mitochondria were stained with MitoTracker‐Red. The mean mitochondrial content and mitochondrial interconnectivity index (Area/Perim) are shown as mean ± *SD*. The drug solvent used was DMSO, and equivalent amounts of DMSO were used in parallel to the drug as a treatment control. Representative confocal images and quantifications are shown. *indicates Mann–Whitney *p* < 0.05, **indicates *p* < 0.01, ***indicates *p* < 0.001. NS, not significant. Scale bars, 10 μm. (g) A mechanistic model of the signaling loop in normal aging and PD pathogenesis is proposed, and the potential drug intervening points are illustrated

In humans, *PINK1*‐deficiency leads to mitochondrial defects (Bueno et al., 2015). To determine whether our findings were relevant to human pathology, we first used human SH‐SY5Y cells to examined the potential impact of *SMARCA4* on *PINK1* depletion. Lower mitochondrial contents and more fragmentated mitochondrial network were observed in the *PINK1* RNAi‐treated SH‐SY5Y cells. While *SMARCA4* RNAi alone did not significantly perturb the mitochondrial content and morphology, the genetic manipulation ameliorated the mitochondrial abnormality caused by *PINK1* RNAi (Figure 6a,b), which was in line with our previous results in *Drosophila*, implicating the potential conserved role of SMARCA4 in the dopaminergic neurons. Furthermore, we generated a *PINK1* ^−/−^ HeLa cell line, using the CRISPR/Cas9 technology (Figure 6c) (Mali et al., 2013). Enhanced phosphorylation of ERK and MEK1 was observed in the *PINK1* ^−/−^ HeLa cells, indicating an aberrant MAPK signaling over‐activation (Figure 6d). Treatment with the MEK1 inhibitor, PD0325901, effectively mitigated multiple mitochondrial defects in the *PINK1* ^−/−^ cells, such as reduced mitochondrial membrane potential, lowered mitochondrial contents, and abnormal network interconnectivity (Figure 6e,f). In agreement with our results, MEK1 inhibitor has been shown to reverse PD‐associated phenotypes induced by pathological *LRRK2* alleles in cultured human iPS derived neurons (Reinhardt et al., 2013).

## 3 DISCUSSION

In summary, we identified a subset of 32 novel PD‐associated genes, which were highly enriched in aging and neural disorders. The roles of two candidate genes SMARCA4/Brm and BLVRA/BVR were validated in vivo. The activity of ERK‐ETS signaling, as a common effector for SMARCA4/Brm and BVR, was found to be also elevated in different genetic forms of *Drosophila* PD models. Thus, we have discovered a potential convergent PD pathogenesis pathway. Our finding also underscores the important role of epigenetic regulators and reveals a novel epigenetic target besides HDACs and DNA methyltransferases (DNMTs) for the therapeutic interventions of aging‐related disorders, including PD. Remarkably, the in vivo genetic manipulations in our studies were specifically restricted in the dopaminergic neurons thanks to the GAL4‐UAS binary expression system in *Drosophila*, highlighting the cell autonomous impacts of the target genes. In fact, genetic modifications of vulnerable dopaminergic neurons per se may be valuable for cell/genetic therapy in PD patients in the future.

Brm was found here to be progressively upregulated in the aging brains of PD fly models. One possibility is through a genetic imprinting response to elevated calcium level triggered by aberrant neuronal activities or calcium metabolism (Zhang et al., 2016). Alternatively, Brm could be activated through NF‐kB mediated inflammatory responses that are well recognized in the development of neurodegenerative diseases (Bonnay et al., 2014). Besides, there is a clue that Brm functions downstream of Hippo pathway and plays an important role in a feedback loop between Crumb and Yorkie in this pathway (Zhu et al., 2015). On the other hand, in our unpublished data, we did observe the differential expression of other oxidoreductases (e.g., P450, sulfiredoxin, and phenoloxidase) at the early‐middle stage of PD. Therefore, it remains possible that Brm might directly interact with Keap1/Nrf to elicit a program of sequential cellular responses to oxidative stress in DA neurons.

Inactivation of Brm was shown here to prevent dopaminergic neurons from degeneration. One putative route was through modulating the MAPK/ERK signally activity. Accordingly, Brm was shown to directly interact with Dsor1 (MEK1) and promotes EGFR‐Ras‐MAPK signaling activity (Friedman et al., 2011; Herr et al., 2010). Nevertheless, the possibility could not be ruled out that direct interactions exist between Brm with transcriptional factors downstream of MAPK/ERK signaling, such as Pnt or Aop, constituting a positive auto‐regulation feedback loop. Alternative mechanisms await further investigation to elucidate the effects of Brm.

Our finding that Brm inactivation protects DA degeneration seems to be at odds with the positive roles of Brm in the neural development (De Rubeis et al., 2014; Koga et al., 2009; Zhang et al., 2016). However, previous studies have shown SWI/SNF complexes in different tissues at different stages have distinct functions by eliciting context‐specific transcription programs. It is very likely that Brm may need to collaborate with other SWI/SNF components and DA neuronal‐specific transcription factors to regulate the expression of target genes in DA neurons. Such possibility can help to explain why we did not observe substantial pathogenic effect by overexpressing Brm in the *Drosophila* DA neurons. Dosage and non‐linear effect might also contribute to the insufficiency. Nevertheless, future works should clarify the complex role of Brm in the aging neurons.

On the other hand, we used the String PPI database to search for BLVRA and SMARCA4, and could not find any direct interaction between the two proteins. Considering that SMARCA4 is an epigenetic factor and BLVRA was shown to interact directly with MEK/ERK and serve as an ERK activator (Lerner‐Marmarosh et al., 2008), we speculate that SMARCA4 might function downstream of BLVRA/MEK/ERK, which is in line with our finding that overexpression of the *Drosophila* BLVRA homologue dBVR alone led to dopaminergic degeneration and aggravated degenerative phenotypes in multiple PD models, which could be remarkably rescued by inactivation of the *Drosophila* homologue of SMARCA4 (Brm). The detail molecular mechanisms how they act in concert to modulate MEK/ERK signaling await further investigations.

Different mitogen‐activated protein kinase (MAPK) pathways have been linked to aging and age‐related disorders, for example, different forms of PD, especially the Jun amino‐terminal kinases (JNK) and p38 MAPK pathways. However, the role of MEK‐ERK is surprisingly elusive and somewhat controversial (Kautu, Carrasquilla, Hicks, Caldwell, & Caldwell, 2013; Kim & Choi, 2010; Reinhardt et al., 2013), very likely owing to the fact that most previous studies were performed in vitro resulting in contradictory results. Our results thus clarify the hitherto controversial role of MEK‐ERK activation in age‐related PD pathogenesis by concluding that chronic and prolonged over‐activation of MEK‐ERK beyond a beneficial range results in DA neurotoxicity.

Previously, targeted inhibition of ERK signaling was demonstrated to mitigate spinocerebellar ataxia type 1 in *Drosophila* and mice models (Park et al., 2013). In addition, direct attenuation of MAPK/ERK was shown to be sufficient to extend life span of *Drosophila* (Slack et al., 2015). Indeed, three compounds that inhibited MEK‐ERK signaling also ameliorated DA degeneration in all PD fly models in current studies. Among them, Trametinib, an FDA‐approved drug for the treatment of melanoma, has been shown to extend life span (Slack et al., 2015). Our finding thus adds another piece of evidence that MAPK/ERK signaling could be a crucial intervention for aging and age‐related disorders. Nevertheless, different tissues or cellular contexts might differentially respond to distinct level of MAPK/ERK, and the understanding of the quantitative relationship between ERK signaling and outcomes would benefit future efficacy study of ERK inhibitors upon life span and age‐related disorders.

To our knowledge, this manuscript represents the first report of compounds that are effective in preventing DA degeneration in vivo in the four most common genetic forms of PD, compared to a previous report of Rapamycin (Tain et al., 2009). MEK‐ERK‐ETS and mTOR/4E‐BP pathways might converge on common downstream effectors such as mitochondrial activity/quality control, proteostasis, autophagy, oxidative stress response, DNA damage repair, and DNA‐chromatin modifications, all of which are important aspects during aging. It is conceivable that cocktail strategies using both lines of inhibitors might eventually balance symptoms mitigation and side effects. Due to the high degree of evolutionary conservation in the SWI/SNF, BLVR/HO, and MEK‐ERK‐ETS pathways, our study may reveal multiple therapeutic entry points to reverse DA degeneration, delay the aging of organs (such as brain, skin, kidney and etc.) and extend the life span in humans (Figure 6g).

## 4 EXPERIMENTAL PROCEDURES

### 4.1 Frequent gene co‐expression analysis

Eleven gene expression datasets from NCBI Gene Expression Omnibus (GEO) were used: GDS2519, GDS2821, GDS3128, GDS3129, GSE19587, GSE20141, GSE20146, GSE20153, GSE20292, GSE20295, and GSE20333. Detailed bioinformatics analysis is described in Appendix S1.

### 4.2 SNP query method

PD gene database PDGene (http://www.pdgene.org) was used (Nalls et al., 2014), detail in the Appendix S1.

### 4.3 *Drosophila* Stocks and nomenclature

Fly genotypes for each experiment were listed in the Appendix S2. Detail information of fly strains and husbandry was described in the Appendix S1.

### 4.4 *Drosophila* lifespan assays

Adult survival curves were constructed at 29°C by using previously described methods (Flatt & Kawecki, 2007; Parmar & Machin, 1995), which is described in the Appendix S1.

### 4.5 *Drosophila* PD models and pathologic phenotype evaluation

At least 20 hemisphere brains were quantified via double‐blinded fashion for each data point (*n* > 20), details in the Appendix S1.

### 4.6 Semi‐quantitative RT‐PCR and Quantitative Real‐time PCR

Details in the Appendix S1.

### 4.7 Whole‐mount Brain Immunostaining, live imaging, and Microscopy

All images were taken by a confocal microscopy (Leica TCS SP5) with identical instrument parameters for any given individual experimental series, details in the Appendix S1.

### 4.8 Western Blot Analysis

Details in the Appendix S1.

### 4.9 Pharmacologic treatment experiment

U0126 [1,4‐Diamino‐2,3‐dicyano‐1,4‐bis (o‐aminophenylmercapto) butadiene, Selleck #S1102), PD0325901 (Selleck #S1036) or Trametinib (Selleck #S2673) were applied. See Appendix S1 for details.

### 4.10 Generation of *PINK1* knockout HeLa cell lines using CRISPR/Cas9 gene editing

See Appendix S1 for details.

### 4.11 Assessment of mitochondrial membrane potential (MMP)

Mitochondrial membrane potential was assessed with the probe JC‐1 (Invitrogen). See Appendix S1 for details.

### 4.12 Assessment of mitochondrial content and morphology

The mitochondrial content and morphology were assessed with Mito‐Morphology Macro in ImageJ as previously described (Dagda et al., 2009; Schneider, Rasband, & Eliceiri, 2012).

### 4.13 Genes knockdown in SH‐SY5Y cells

Three pairs of siRNAs target to human PINK1 or SMARCA4 were designed and synthetized. SH‐SY5Y cells were transfected with siRNAs to perform mitochondrial content and morphology analysis.

### 4.14 Assessment of whole brain Redox state

The CM‐H2 DCFDA fluorescein dye (Invitrogen, Cat# C400) and redox‐sensitive GFPs (roGFPs) protein, fly lines of tub‐mito‐roGFP2 or UAS‐roGFP2 genotype were employed to measure the whole brain ROS stress of PD *Drosophila*. (Liu, Celotto, Romero, Wipf, & Palladino, 2012; Wu, Cao, Chang, & Juang, 2017).

### 4.15 Quantification and statistical analysis

Mann–Whitney test or log‐rank test was used, respectively. See details in the Appendix S1.

## CONFLICT OF INTEREST

The authors declare no competing financial interests with patent applications having been filed relating to this work.

## AUTHOR CONTRIBUTIONS

Y.Y. conceived the project. Y.Y. and K.H. designed and supervised the experiments. J.Z. and K.H. performed gene co‐expression analysis, PPI analysis, and SNP query. Y.Y., L.S., Y.C., W.C., Y.Z., Y.Y., M.X., F.M., Y.R., F.C., M.E.H, S.N., Z.Y., Z.S., and X.Z. performed NCBI GEO dataset search, fly genetics, immunohistochemistry, and imaging experiments. L.S. and X.Z. performed p‐element constructions and microinjections. L.S, Y.Z., Y.C., X.Z. and H.Z. performed Western blotting and drug treatment experiments. W.C., Z.Z., M.Y., Y.Y., M.Y., and K.C. performed human cells studies. Y.Y., L.S., J.Z., and K.H. wrote the manuscript, with contributions from other co‐authors.

## Supporting information

Table S5

Supplementary Material

## Data Availability

The data that support the findings of this study are openly available in Mendeley Data at http://dx.doi.org/10.17632/6587ksptsf.1.
