# Supplementary material for: Attenuation of epigenetic regulator SMARCA4 and ERK‐ETS signaling suppresses aging‐related dopaminergic degeneration
Source: Aging Cell. 2020 Aug 4;19(9):e13210. doi: 10.1111/acel.13210 (PMC7511865; doi:10.1111/acel.13210)
Supplement: Supplementary file 2 — Supplementary Material [file ACEL-19-e13210-s002.docx]

**Supplementary Materials and Methods**

**Frequent gene co-expression analysis**

We chose seven of the most commonly known PD genes as anchor genes. They are ATP13A2, HTR2A, SCNA, LRRK2, PARK2, PARK7, and PINK1. In addition, we identified eleven gene expression datasets from NCBI Gene Expression Omnibus (GEO), which contain samples from human brain tissues, especially the *substantia nigra* region in which the death of dopamine contain cells leads to PD. They are: GDS2519, GDS2821, GDS3128, GDS3129, GSE19587, GSE20141, GSE20146, GSE20153, GSE20292, GSE20295, and GSE20333.

Our workflow is similar to previously described in Conference papers, with a slight modification as in the following steps:

Step 1: For the i-th dataset (i= 1, 2, …, 11), compute the Pearson correlation coefficients (PCC) between every pair of genes within each dataset, and set the top five percentile of all PCC values as threshold Ti. PCC values were converted to absolute values before setting the threshold.

Step 2: For the k-th anchor gene Ak (k = 1, …, 7), denote as the set of genes in the i-th dataset whose PCC values with respect to Ak are higher than Ti. These genes are considered to have high correlation with Ak in the i-th dataset.


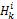

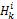


Step 3: For a gene Gj, its frequency of having high correlation with Ak is denoted as

,

where is the indicator function which is 1 if the input is TRUE and 0 otherwise.


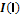

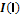


Step 4: For each anchor gene Ak, let Pk be the set of genes with high values. Specifically,


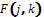

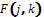


which is the collection of all genes that have high PCC values with Ak in at least five datasets for all seven anchor genes.

Step 5: Finally, for every gene Gj, count the frequency that it appears in Pk (k =1, ..., 7). The count number of each gene Gj can be derived as

Finally, the selected gene networks are subjected to gene set enrichment analysis using TOPPGene (<http://toppgene.cchmc.org/>) and Ingenuity Pathway Analysis (IPA®, <http://www.ingenuity.com>).

The 32 PD candidate gene list is the result of a very stringent selection process, which involves three thresholds for gene selection. First, in each dataset only genes with top 5-percentile correlation coefficients for a specific anchor gene is selected for the next step. Second, only genes who have strong correlation (within top 5-percentile) with at least three anchor genes (out of total of seven anchor genes) are further selected as the gene list for this dataset. Third, genes that are selected in at least five datasets out of a total of 11 datasets are used in this study. Statistical analysis (based on Fisher’s exact test) shows that the chance for a random gene to be selected through this process is 3.4E-10.

**SNP query method**

We queried with gene symbol “SMARCA4” and “BLVRA” in PD gene database PDGene ([http://www.pdgene.org](http://pdgene.org)), which described that the SNP data source as “the data” currently available on PDGene include all results pertaining to the discovery phase of the GWAS meta-analysis by ([Nalls et al., 2014](#_ENREF_5)). This includes data on 7,782,514 genetic variants in up to 13,708 PD cases and 95,282 controls from 15 independent GWAS datasets of European descent. Variants were imputed using the August 2010 release of the 1000 Genomes Project European-ancestry haplotype reference set and filtered according to standard quality control criteria. In line with the criteria applied in the published study ([Nalls et al., 2014](#_ENREF_5)), only variants with a minor allele frequency ≥0.1% and those assessed in at least 3 of the 15 datasets have been included for display in PDGene. The database also includes association results of genotyping data generated on the “NeuroX chip”, a semi-custom genotyping array, on 5,353 PD cases and 5,551 controls of European descent for the most significantly associated polymorphisms from the discovery phase (i.e. across 26 loci showing genome-wide significant association (p <5x10^-8^) in the discovery phase with PD risk) as well as for 6 additional, previously reported GWAS signals. Details on the included datasets as well as all genotyping procedures and statistical analyses can be found in our original publication ([Nalls et al., 2014](#_ENREF_5)).

***Drosophila* Stocks and nomenclature**

Fly strains were maintained with standard molasses-cornmeal-yeast food and were kept in 25°Cwhereas PD model flies were maintained at 21-25°C routinely and raised at 29°C for the experiments unless mentioned. TH-Gal4 was a gift from S. Birman ([Friggi-Grelin et al., 2003](#_ENREF_3)). Brm::GFP is the Bloomington stock #59784. UAS-dBVR plasmid was derived from fly cDNA and molecular cloning, and the corresponding p-element mediated transgenic flies were generated by standard microinjection protocol using *w^1118^* stock. Two UAS-dBVR transgenic lines were used in this study, UAS-dBvr and UAS-dBvr (III). Pnt::EGFP is the Bloomington stock #42680 or #60528. UAS-Brm wt, UAS-Brm^DN^, UAS-Brm RNAi fly strains were gifts from Helena E. Richardson. UAS-Lrrk2^I1915T^ and UAS-Pink1 RNAi fly strains were gifts from Bingwei Lu ([Wang et al., 2006](#_ENREF_9)). UAS-αSynA30P was a gift from Nancy Bonini ([Warrick et al., 1998](#_ENREF_10)). The *pink1^B9^* fly strain was a gift from Jongkyeong Chung. Parkin RNAi is the Bloomington stock #38333. GstD-GFP was a gift from Dirk Bohmann. Other fly strains were obtained from Bloomington *Drosophila* Stock Center (#31603, #33639, #34855, #35038, #869, #5789, #5790, #34909, #59006, #67672) and Vienna *Drosophila* Research Center (dBVR RNAi, v24042). The age of adult flies was defined as the days after eclosion (AE). Further information on genes and symbols can be found in Flybase (http://ﬂybase.bio.indiana.edu). Fly genotypes for each experiment were listed in the supplementary appendix.

***Drosophila* Lifespan Assays**

Adult survival curves were constructed at 29°C by using previously described methods ([Flatt & Kawecki, 2007](#_ENREF_2); [Parmar & Machin, 1995](#_ENREF_6)). Briefly, within a 24-hour eclosion period, an average of 30 adult flies were introduced into a fly-medium vial for each experiment. Dead individuals were recorded and removed every 3 days, and fly-medium vial was changed. Vials were maintained on a 12 h:12 h light:dark cycle. Three to five replicate vials were used for each genotype. Survival data were pooled across replicate vials for each genotype. Survival rate (fraction of flies alive, *lx*) was estimated as N_X_/N_0_, where N_X_ is the number of flies alive at the beginning of each census interval and N_0_ is the initial cohort size ([Parmar & Machin, 1995](#_ENREF_6)). At least two cohorts for each genotype were surveyed and the average N_X_/N_0_ were used to construct the survival curves. Data were analyzed with PraphPad Prism 7.0 and survivorships between genotypes were compared and tested for significance with a log-rank test (significant difference at P < 0.05).

***Drosophila* PD models and pathologic phenotype evaluation**

The following genotype-based *Drosophila* PD models were used in this study: 1) TH-Gal4>UAS-αSynA30P; 2) TH-Gal4>UAS-Lrrk2^I1915T^; 3) TH-Gal4>UAS-Parkin RNAi (Bloomington #38333); 4) *pink1*^B9^; TH-Gal4 or TH-Gal4>UAS-PINK1 RNAi. These four *Drosophila* PD models were abbreviated as αSyn, Lrrk2, Parkin, Pink1 (Pink1 mut and Pink1 RNAi) PD models respectively. Control flies were TH-Gal4>w- or TH-Gal4>UAS-Luc RNAi. Homozygous *parkin* null alleles were found not healthy, so they were not used for PD modeling in this study. Parkin RNAi flies were used instead. *pink1*^B9^ was primarily used as the *pink1*-related PD fly model, see genotype listed in the supplementary appendix. Male flies were used unless mentioned. Experimental flies were sorted into individual vials at a density of 15~20 flies per vial and were transferred to fresh vials three times a week. Experimental flies were raised at 29°C. DA neurons were marked by anti-tyrosine hydroxylase (TH) antibody. The DA neuron number in the lateral protocerebral posterior 1 (PPL1) cluster was scored. Left and right PPL1 clusters in individual fly brains were scored independently. Only well-dissected, processed, mounted and preserved fly brains were used for the quantifications. The intact left or right PPL clusters contains 11~12 DA neurons in healthy individuals. 2-day-old (or 2^nd^ day AE) and 30-day-old (or 30^th^ day AE) adult flies were subjected to evaluation of DA neurons for most experiments unless noted. At least 20 hemisphere brains were quantified via double-blinded fashion for each data point (n>20). Two biological replicates were carried out.

**Semi-quantitative RT-PCR and Quantitative Real-time PCR**

Total RNA extracted from fly heads with RNA extraction kit (TRIZOL reagent, Invitrogen, Inc.) was used for semi-quantitative reverse transcription-PCR (Sq-RT PCR) and Quantitative real-time PCR (Q-RT PCR). The cDNA was synthesized using the Reverse Aid First strand cDNA synthesis kit (Thermo Fisher, Cat NO: K1622). Specific primer pairs were used for BVR and αSYN gene expression analysis. Expression levels of any given genes were resolved by agarose gel electrophoresis for the Semi-quantitative RT-PCR. The samples were analysis by Quantity One BioSoft (BIO-RAD). Relative expression levels were normalized to that of tubulin. Two biological replications were carried out for each sample. The Q-RT PCR was performed using a LightCycler® 96 System instrument with Ultra SYBR Mixture (CW Bio, CW0957M). The data display fold change relative to the control after normalization to a-Tubulin. More than three replications were carried out.

**Whole-mount Brain Immunostaining, live imaging and Microscopy**

Adult flies were collected for brain dissection at the indicated time points. Brains were fixed for 2 hours in 4% buffered formaldehyde at 25°C, washed in phosphate-buffered saline (pH 7.4) with 0.2% Triton X-100 (PBT), blocked in 5% goat serum in PBT (PBST) for 30min at room temperature and incubated in primary antibody overnight at 4°C. Primary antibodies were prepared in blocking buffer solution (Rabbit anti-TH, Millipore, #AB152). After three times of 15-min-wash, brains were incubated with goat anti-rabbit secondary antibodies (Life AlexaFluo® 488, 568) for 2 h at room temperature, followed by thorough rinses and mounted. For live imaging, fly brains or imaginal discs were promptly dissected in *Schneider*’s insect medium (Life-Gibco), properly mounted and scanned, stained with Hoechst33342 (Beyotime) in some cases prior to imaging in some cases by standard protocol. Average fluorescence intensity of Brm::GFP, Pnt::EGFP or GstD-GFP was quantified with Metamorph software (Leica AF lite), normalized to those in control flies. At least five well-preserved fly brains were used for fluorescence intensity quantifications. All images were taken by a confocal microscopy (Leica TCS SP5) with identical instrument parameters for any given individual experimental series. Images were processed with Adobe Photoshop and subjected to identical post-acquisition brightness/contrast effects.

**Western Blot Analysis**

Fly heads were homogenized with a pestle, and protein extracts were prepared with lysis buffer (20 mM Tris/HCl at pH 7.6, 150 mM NaCl, 5mM EDTA, 10% glycerol, 1% SDS and 1 mM PMSF). Supernatants were collected after 16000g and 4°C for 10 min, added with 5×SDS loading buffer and boiled for 5 min at 95°C. For immune-blotting analysis, protein lysates were electrophoresed with SDS-PAGE and transferred to PVDF membranes (Bio-Rad). Membranes were blocked in 5% BSA in PBS-T, followed incubation with diluted antibodies and secondary antibodies in blocking solution. Primary antibodies used were: monoclonal mouse anti-alpha tubulin (DSHB), monoclonal mouse anti-pERK (Sigma #M8159), rabbit anti-ERK (Cell Signalling #4695). Proteins were visualized using the Immobilon Western chemiluminescent HRP substrate (Beyotime) on ChemiDoc^TM^ XRS+ (BIO-RAD). Band intensity was calculated and analyzed with the Quantity One v4.62 (BIO-RAD). At least three biological replicates were performed and their means were calculated. Statistics were analyzed with Student’s *t*-test for numerical data.

HeLa cell lines were grown in Dulbecco’s Modified Eagle Medium (DMEM) containing 10% fetal bovine serum in a 5% carbon dioxide (CO_2_) atmosphere. Control and *pink1*-KO HeLa cells were homogenized with RIPA Lysis Buffer (Beyotime). Blots were probed with the following antibodies: monoclonal mouse anti-alpha tubulin (DSHB), rabbit anti-PINK1 (Cell Signalling #6946), monoclonal mouse anti-pERK (Sigma #M8159), rabbit anti-ERK (Cell Signalling #4695), rabbit anti-pMEK (Cell Signalling #3958), rabbit anti-MEK (Cell Signalling #13033).

**Pharmacologic treatment experiment**

The experimental flies were collected after eclosion, assorted into 20 flies per via and raised at 29°C for drug treatment. U0126 [1,4-Diamino-2,3-dicyano-1,4-bis (o-aminophenylmercapto) butadiene, Selleck #S1102) were dissolved with DMSO in the recommended stock solution (10 mg/mL). Flies were fed for 4h with a serial concentration gradient of U0126, 10 μg/mL and 1 μg/mL, which were diluted with 4% sugar water, and were transferred back to standard fly food after drug exposure. Prior to drug treatment, a food dye supplement was used to justify the feasibility of ingestion of drugs by flies via this protocol. Drug treatment was performed continuously in a 24h cycle until the flies were harvested for protein analysis (7 days drug treatment) or whole-mount immunostaining analysis (30 days drug treatments). For control treatments, equivalent volumes of the vehicle alone were added. The application of the PD0325901 (Selleck #S1036) or Trametinib (Selleck #S2673) followed the identical procedure as U0126, with 1 μg/mL and 10 μg/mL feeding concentration for PD0325901, and 1.624 μM and 16.24 μM for Trametinib, respectivefully. Two independent sets of biological experiments were performed.

**Generation of *pink1* knockout HeLa cell lines using CRISPR/Cas9 gene editing**

The HeLa cell line was sent to GENEWIZ, Inc. (Beijing, China) to perform authentication test. Firstly, genomic DNA was extracted from the cell pellets. Samples together with positive and negative control were amplified using GenePrint 10 System (Promega). Then, the amplified products were processed using the ABI3730xl Genetic Analyzer. Finally, data were analyzed using GeneMapper software V.4.0 and then compared with the ATCC for reference matching. To generate *pink1* knockout cell lines, CRISPR guide RNAs (gRNAs) were chosen to target exon 1 which is common to all splicing variants. Oligo nucleotides containing CRISPR target sequences (5’-CCGGCCGGGCCTACGGCTTG-3’) were annealed and ligated into pSpCas9 (BB)-2A-GFP (PX458) (Addgene 48138). Then, HeLa cells were transfected with this Cas9-2A-GFP and gRNA constructs. Two days after transfection, DNA from polled cells were extracted and the targeted genomic regions were PCR amplified. PCR products were subjected to Sanger sequencing analysis to verify the potential success of targeting. GFP-positive cells were sorted by FACS and plated in 96-well plates. Single colonies were expanded for depletion screening of the mutations. Knockout lines were further confirmed by Sanger sequencing. A cell clone harboring two heterogeneous frame-shift mutations at the *pink1* locus was used for subsequent experiments, referred as *pink1^-/-^*. Western blot analysis was conducted to validate the loss of Pink1 with the anti-PINK1 (D8G3) Rabbit mAb (Cell Signaling, #6946). DMSO was the solvent and equivalent amount was used in parallel as the drug treatment control.

**Assessment of mitochondrial membrane potential (MMP)**

Mitochondrial membrane potential was assessed in WT and *pink1*-KO HeLa cells with the probe JC-1 (Invitrogen). JC-1 accumulates within the intact mitochondria to form multimer J-aggregates that result in a shift of fluorescence from green (530 nm) to red (590 nm). The potential-sensitive color shift is due to concentration-dependent formation of red fluorescent J-aggregates. A change of fluorescence from red to green indicates decreased MMP. Cells were treated with PD0325901 (50 nM), a selective and non ATP-competitive MEK inhibitor, for 8 hours. Then, the cells were loaded with 5 μg/ml of JC-1 for 3 minutes at 37°C. The cells were rinsed with phosphate-buffered saline, and mitochondrial JC-1 was analyzed by a Leica SP5 confocal microscopy and the Leica MetaMorph software. More than 30 randomly selected individual cells were analyzed for each data points. Three biological replicates were performed.

**Assessment of mitochondrial content and morphology**

Mitochondrial content and morphology in WT and *pink1*-KO HeLa cells were visualized with Mito-Tracker Red (Molecular Probes) by a Leica SP5 confocal microscopy. Cells were treated with PD0325901 (50 nM) for 8 hours and then stained with MitoTracker for 30 min. Finally, the mitochondrial content and morphology were assessed with Mito-Morphology Macro in ImageJ as previously described ([Dagda et al., 2009](#_ENREF_1); [Schneider, Rasband, & Eliceiri, 2012](#_ENREF_7)). DMSO was the solvent and equivalent amount was used in parallel as the drug treatment control. More than 30 randomly selected individual cells were analyzed for each data points. Three biological replicates were performed.

**Genes knockdown and mitochondrial assessment in SH-SY5Y cells**

SH-SY5Y cells were cultured in a 1:1 mixture of ATCC-formulated Eagle's Minimum Essential Medium, Catalog No. 30-2003, and F12 Medium supplemented with 10% FBS (Hyclone), 100U/mL penicillin-streptomycin. Three pairs of siRNAs target to human Pink1 or SMARCA4 were designed and synthetized by Sangon Biotech (Shanghai) Co., Ltd. One negative control (NC) was also synthetized. Each siRNA was dissolved in DEPC H2O to make 20 µM stock solution. The sense sequences of siRNAs were shown below:

siPink1-1: 5’-CGGACGCUGUUCCUCGUUAUGTT-3’

siPink1-2: 5’-GCAGCGUAGCAUGUCUGAUUUTT-3’

siPink1-3: 5’-GAAGCCAUCUUGAACACAAUGTT-3’

siSMARCA4-1: 5’-GUACCGAGCCUCGGGUAAAUUTT-3’

siSMARCA4-2: 5’-CAAGAUGUCGAUGAUGAAUAUTT-3’

siSMARCA4-3: 5’-CGGCAGACACUGUGAUCAUUUTT-3’

NC: 5’-UUCUCCGAACGUGUCACGUTT-3’

For each target, 6 µL of siRNAs (2 µL of each) plus 2 µg of pCDNA3.1-EGFP plasmid (MiaoLing Plasmid Sharing Platform, P0158) were transfected using electroporator from Celetrix biotechnologies. After 24 hours, total RNA was extracted with TRIGene (GenStar, P118). The cDNA was synthesized using the HiScript III RT SuperMix for qPCR (+gDNA wiper) (Vazyme, R323). qPCR was performed with RealStar Green Fast Mixture (GenStar, A301). The following primers were used: ACTB (5ʹ-CATGTACGTTGCTATCCAGGC-3’ and 5ʹ-CTCCTTAATGTCACGCACGAT-3’); GAPDH (5ʹ-CTGGGCTACACTGAGCACC-3’ and 5ʹ-AAGTGGTCGTTGAGGGCAATG-3’); Pink1 (5ʹ-CCCAAGCAACTAGCCCCTC-3’ and 5ʹ-GGCAGCACATCAGGGTAGTC-3’); SMARCA4 (5ʹ- AATGCCAAGCAAGATGTCGAT-3’ and 5ʹ- GTTTGAGGACACCATTGACCATA-3’). Mitochondrial content and morphology were monitored as it did in the HeLa cells.

**Assessment of whole brain Redox state**

The CM-H2 DCFDA fluorescein dye (Invitrogen, Cat# C400) and redox-sensitive [GFPs](https://www.sciencedirect.com/topics/neuroscience/green-fluorescent-protein) (roGFPs) protein were employed to measure the whole brain ROS stress of PD *Drosophila*. Measurement methods involved in were introduced from previously reported publications ([Liu, Celotto, Romero, Wipf, & Palladino, 2012](#_ENREF_4); [Wu, Cao, Chang, & Juang, 2017](#_ENREF_11)). The *Drosophila* brains with different genetic backgrounds were live dissected and incubated with 10 μM DCFDA for 5 min at RT if applicable, brain images were captured using a Leica TCS SP5 II confocal microscope with 488 nm excitation and 525 nm emission. Images were analyzed by image J software.

Alternatively, fly lines of tub-mito-roGFP2 or UAS-roGFP2 genotype were crossed with the PD models respectively in order to estimate the ROS level in whole brain or in the PPL1 neurons. The aged *Drosophila* brains were dissected in PBS with 20 mM NEM (N-ethyl maleimide) and then were imaged with a 535nm filter, followed with excitations at 405 nm and 488 nm. Image J software were employed to analyzed the 405 nm:488 nm ratios.

**Quantification and statistical analysis**

Error bars represent standard deviations (S.D.) as indicated in the figure legends. Statistical analyses were performed using GraphPad Software (GraphPad Prism 7.0). For lifespan assay, survivorships between genotypes were compared through survival curves and tested for significance with a log-rank test (significant difference at P < 0.05). For other assays, statistical analysis of differences between two groups was performed using Mann-whitney test. * indicates P < 0.05; **, P < 0.01; ***, P < 0.001; ns, not significant. Differences were considered statistically significant at P < 0.05.

**Supplementary Figure Legends**

**Supplementary Figure S1:**

The subcellular localization of Brm::GFP. (a–c) Representative live images of eye discs (a), wing discs (b) and salivary glands (c) from Brm::GFP larvae. Nuclei were stained with Hoechst in blue. Scale bar, 10 μm.

**Supplementary Figure S2:**

Characterization pan-neuronal overexpression or knockdown of dBVR in *Drosophila*. (a, b) Semi qPCR experiments verified the RNAi and overexpression effects of dBVR. *elav*-Gal4 was used as the driver line. Representative electrophoresis image (a) and the quantifications (b) were shown. Tubulin served as the internal control. Mean ± SEM was shown (n≥3). * indicates two-tails Student’s t-test with *P* < 0.05. (c, d) Pan-neuronal overexpression but not RNAi of dBVR led to progressive PPL1 DA neuronal loss. Scoring PPL1 DA neurons in 2-day-old (c) and 35-day-old (d) flies subjected to dBVR-related genetic manipulations. Genetic manipulations included *elav*-Gal4 driving overexpression of wide-type dBVR (UAS-Bvr II or UAS-Bvr III) and induction of BVR RNAi with w- (elav-Gal4/+) and mitodsRed RNAi (elav-Gal4/UAS-mitodsRed RNAi) flies as the control, respectively. n>20 for each data point. ** indicates Mann-whitney with *P*<0.01. NS, not significant.

**Supplementary Figure S3:**

Quantitative RT-PCR analysis of dBVR and αSYN to exclude the potential titration problem of UAS-Gal4 system. (a, b) Analysis of dBVR expression level in the adult fly brains. Genetic manipulations included TH-Gal4 driving overexpression of αSYN, Bvr III, αSYN +Bvr III, αSYN + Bvr III + Aop^wt^, Bvr II, αSYN + Bvr II, αSYN + Bvr II + Brm RNAi, αSYN + Bvr II + Brm^DN^, αSYN + Bvr II + MEK RNAi (a) and TH-Gal4 driving overexpression of Bvr III + Aop^wt^, Bvr II + Brm RNAi, Bvr II + Brm^DN^, Bvr II + Brm^GOF^, Bvr II + MEK RNAi (b). Mean ± SEM (n≥3). (c, d) Analysis of αSYN expression level in the adult fly brains. Genetic manipulations included TH-Gal4 driving overexpression of αSYN, αSYN + Brm RNAi, αSYN + Brm^DN^ (c) and TH-Gal4 driving overexpression of αSYN, αSYN + Bvr III, αSYN + Bvr III + Aop^wt^, αSYN + Bvr II, αSYN + Bvr II + MEK RNAi, αSYN + Bvr II + Brm RNAi, αSYN + Bvr II + Brm^DN^ (d). Mean ± SEM was shown (n≥3). ** indicate Mann-whitney with *P* < 0.01. NS, not significant.

**Supplementary Figure S4:**

Establishment of four *Drosophila* PD models. (a, b) Degenerative DA neuronal loss in PPL1 clusters in four *Drosophila* PD models, abbreviated as αSyn, Lrrk2, Parkin, Pink1 (Pink1 null mutant [Pink1 Mut] and Pink1 RNAi) PD models respectively. Control flies were TH-Gal4>w-. PPL1 DA neurons that were marked by anti-tyrosine hydroxylase (TH) antibody were counted. 2-day old (a) and 30-day-old (b) adult flies were used for analysis, n > 20 for each data point. The genotypes for experimental flies are provided in the supplementary appendix. PPL1 DA neurons were reduced in number in 30-day-old PD flies (αSyn, 10.08±0.28; Lrrk2, 10.28±0.45; Pink1 RNAi, 10.54±0.5; Pink1 mut, 9.57±0.82; and Parkin, 10.33±0.47) compared with age-matched controls (11.77±0.42). ** indicates Mann-whitney with *P* < 0.01.

**Supplementary Figure S5:**

Oxidative stress level indicated DCF-DA in the brains of four PD model flies. (a, b) Representative brain images of control and 10 μM DCF-DA staining brains with or without 100 μM H2O2 were shown (a). Quantification of DCF-DA fluorescent signals (b) in *Drosophila* brains were shown. (c) The DCF-DA staining brains of 5^th^ day AE and 15^th^ day AE in control and four PD model *Drosophila* were analyzed and quantified. Error bars represent the SD. Mann-whitney was performed. ****P*<0.001; ns, not significant. Scale bars, 10 μm.

**Supplementary Figure S6:**

Oxidative stress level indicated by tub-mito-roGFP2 in the brains of four PD model flies. The alphaTub84B regulatory sequences were used to control the pan-expression of mito-roGFP2 in the transgenic reporter flies. Mito-roGFP2 is an oxidant receptor peroxidase-based, mitochondria-localized, fluorescent sensor of hydrogen peroxide oxidation. The oxidative stress was indicated by ratio 405/488 nm of roGFP2 signal. Red fluorescent protein (RFP) was used to label DA neurons. Representative whole-mount fluorescence images of control and PD model fly brains of 5^th^ day AE (a) and 15^th^ day AE (b) were shown. At least 10 samples were quantified in each experimental group. *** indicates Mann-whitney with *P* < 0.001, NS means ‘not significant’. Scale bar, 10 μm.

**Supplementary Figure S7:**

Oxidative stress level indicated by UAS-roGFP2 in the PPL1 neurons of four PD model flies. The fluorescence images show roGFP2 (blue and yellow) fluorescence of PPL1 neurons in fly brains of 5^th^ day AE (a) and 15^th^ day AE (b). Red fluorescent protein (RFP) was used to label DA neurons. The oxidative stress of PPL1 neurons indicated by ratio 405/488 nm of roGFP2 signal were analyzed and quantified in control and four PD mode flies. At least 10 samples were quantified in each experimental group. *** indicates Mann-whitney with *P* < 0.001, NS means ‘not significant’. Scale bar, 10 μm.

**Supplementary Figure S8:**

Anti-oxidant response level indicated by GstD-GFP in the brains of four PD model flies. The genomic sequence upstream of the GstD1 gene was used to control the expression of GFP in the transgenic reporter flies ([Sykiotis & Bohmann, 2008](#_ENREF_8)). The transcriptional activity of the GstD enhancer indicated by GFP signal can be induced by oxidants and thus can be served an index of oxidative stress. Red fluorescent protein (RFP) was used to label DA neurons. (a, b) Representative whole-mount fluorescence images of control and PD model fly brains of 10^th^ day AE (a) and 20^th^ day AE (b) were shown. GstD-GFP signal intensity within the DA region (c, d), outside the DA region (e, f) and brain-wide (g, h) were quantified (n > 5). * indicates two tails Mann-whitney with *P*<0.05, NS means“not significant”. Scale bar, 10 μm.

**Supplementary Figure S9:**

Genetic manipulation of Brahma or BVR modulated dopaminergic degeneration in *Drosophila*. (a) Scoring PPL1 DA neurons in 2-day-old flies subjected to Brm-related genetic manipulations. Genetic manipulations included TH-Gal4 driving overexpression of wide-type Brm (Brm wt), a dominant-negative form of Brm (Brm^DN^) and induction of Brm RNAi with w- and Luc RNAi flies as the control. n > 20 for each data point. (b) Scoring PPL1 DA neurons in 2-day-old flies subjected to dBVR-related genetic manipulations. Genetic manipulations included TH-Gal4 driving overexpression of wide-type BVR (Bvr II or Bvr III), Bvr II + Brm RNAi, Bvr II + Brm^DN^, Bvr II + MEK RNAi, Bvr III + Aop^wt^ and induction of BVR RNAi with w- and Luc RNAi flies as the control. n>20 for each data point.

**Supplementary Figure S10:**

Aop*^[wt]^* overexpression prevented DA degeneration caused by dBVR overexpression. Genetic manipulations included TH-Gal4 driving overexpression of Bvr II + MEK RNAi, Bvr III + Aop^wt^ with w- and Luc RNAi flies as the control, respectively. PPL1 DA neurons in 2-day-old (a) and 30-day-old (b) flies were scored for the phenotype. n > 20 for each data point. * indicates Mann-whitney with *P*<0.05. NS, not significant.

**Supplementary Figure S11:**

Genetic manipulation of MEK-ERK-ETS signaling axis modulated dopaminergic degeneration in *Drosophila*. (a) Results of DA neuron-specific genetic manipulations of MEK, ERK, Pnt and Aop. Genetic manipulations included TH-Gal4 driving induction MEK RNAi, ERK RNAi, Pnt RNAi, Aop RNAi and overexpression of Aop, with w- and Luc RNAi flies as the control. PPL1 DA neurons in 30-day-old flies were scored for the phenotype. (b) Scoring PPL1 DA neurons in 2-day-old flies subjected to MEK or ERK RNAi genetic manipulations with w- and Luc RNAi flies as the control. (c) Scoring PPL1 DA neurons in 2-day-old flies subjected to Pnt/Aop-related genetic manipulations. Genetic manipulations included TH-Gal4 driving overexpression of wide-type Aop (Aop^wt^), induction of Pnt RNAi and Aop RNAi, with w- and Luc RNAi flies as the control. n > 20 for each data point. *indicates two tails Mann-whitney with *P*<0.05.

**Supplementary Figure S12:**

Efficacy of MEK1 inhibitors in fly brains delivered by oral administration. (a, b) Inhibitory effect of orally delivered U0126 upon fly brains was validated. Two concentrations of U0126 were applied (L: 1 μg/mL; H: 10 μg/mL). Homogenates of adult fly brains were used for western blot analysis after continuous drug treatment for 7 days. Inactivation of MAPK/ERK was quantified in (b) with beta-tubulin as the input control. (c, d) Effective concentrations of PD0325901 (L: 1 μg/mL; H: 10 μg/mL) or Trametinib (L: 1.6 μM; H: 16 μM) were determined, respectively. DMSO was the solvent and equivalent amount was used in parallel as the drug treatment control. *elav*-Gal4/+ flies were used. Two biological replicates were performed. ** indicates two tails Student’s *t*-test with *P*<0.01, * indicates *P*<0.05.

**Supplementary References**

Dagda, R. K., Cherra, S. J., 3rd, Kulich, S. M., Tandon, A., Park, D., & Chu, C. T. (2009). Loss of PINK1 function promotes mitophagy through effects on oxidative stress and mitochondrial fission. *Journal of Biological Chemistry, 284*(20), 13843-13855. doi:10.1074/jbc.M808515200

Flatt, T., & Kawecki, T. J. (2007). Juvenile hormone as a regulator of the trade-off between reproduction and life span in Drosophila melanogaster. *Evolution, 61*(8), 1980-1991. doi:10.1111/j.1558-5646.2007.00151.x

Friggi-Grelin, F., Coulom, H., Meller, M., Gomez, D., Hirsh, J., & Birman, S. (2003). Targeted gene expression in Drosophila dopaminergic cells using regulatory sequences from tyrosine hydroxylase. *Journal of Neurobiology, 54*(4), 618-627. doi:10.1002/neu.10185

Liu, Z., Celotto, A. M., Romero, G., Wipf, P., & Palladino, M. J. (2012). Genetically encoded redox sensor identifies the role of ROS in degenerative and mitochondrial disease pathogenesis. *Neurobiology of Disease, 45*(1), 362-368. doi:10.1016/j.nbd.2011.08.022

Nalls, M. A., Pankratz, N., Lill, C. M., Do, C. B., Hernandez, D. G., Saad, M., . . . Singleton, A. B. (2014). Large-scale meta-analysis of genome-wide association data identifies six new risk loci for Parkinson's disease. *Nature Genetics, 46*(9), 989-993. doi:10.1038/ng.3043

Parmar, M. K. B., & Machin, D. (1995). Survival analysis: a practical approach. *(Wiley, Chichester, UK)*, P255.

Schneider, C. A., Rasband, W. S., & Eliceiri, K. W. (2012). NIH Image to ImageJ: 25 years of image analysis. *Nature Methods, 9*(7), 671-675.

Sykiotis, G. P., & Bohmann, D. (2008). Keap1/Nrf2 signaling regulates oxidative stress tolerance and lifespan in Drosophila. *Developmental Cell, 14*(1), 76-85. doi:10.1016/j.devcel.2007.12.002

Wang, D., Qian, L., Xiong, H., Liu, J., Neckameyer, W. S., Oldham, S., . . . Zhang, Z. (2006). Antioxidants protect PINK1-dependent dopaminergic neurons in Drosophila. *Proceedings of the National Academy of Sciences of the United States of America, 103*(36), 13520-13525. doi:10.1073/pnas.0604661103

Warrick, J. M., Paulson, H. L., Gray-Board, G. L., Bui, Q. T., Fischbeck, K. H., Pittman, R. N., & Bonini, N. M. (1998). Expanded polyglutamine protein forms nuclear inclusions and causes neural degeneration in Drosophila. *Cell, 93*(6), 939-949.

Wu, S. C., Cao, Z. S., Chang, K. M., & Juang, J. L. (2017). Intestinal microbial dysbiosis aggravates the progression of Alzheimer's disease in Drosophila. *Nat Commun, 8*(1), 24. doi:10.1038/s41467-017-00040-6

**Supplementary Figure S1**


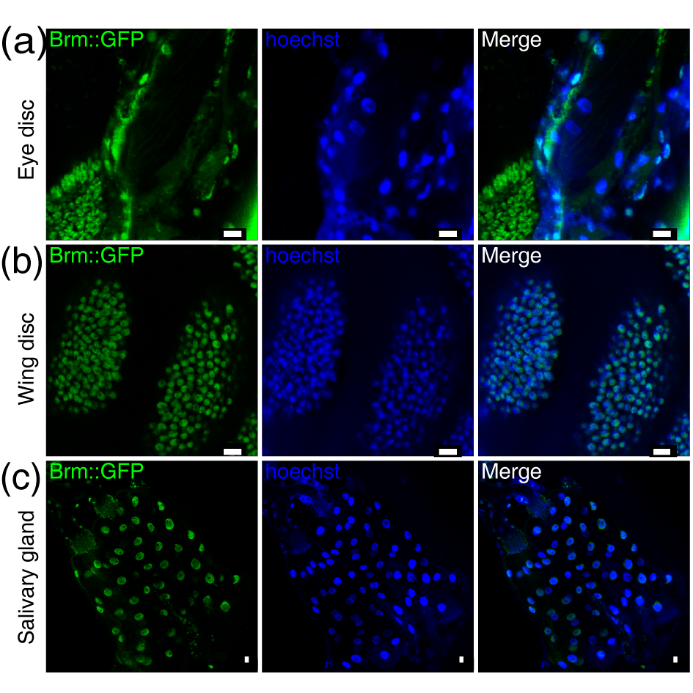


**Supplementary Figure S2**


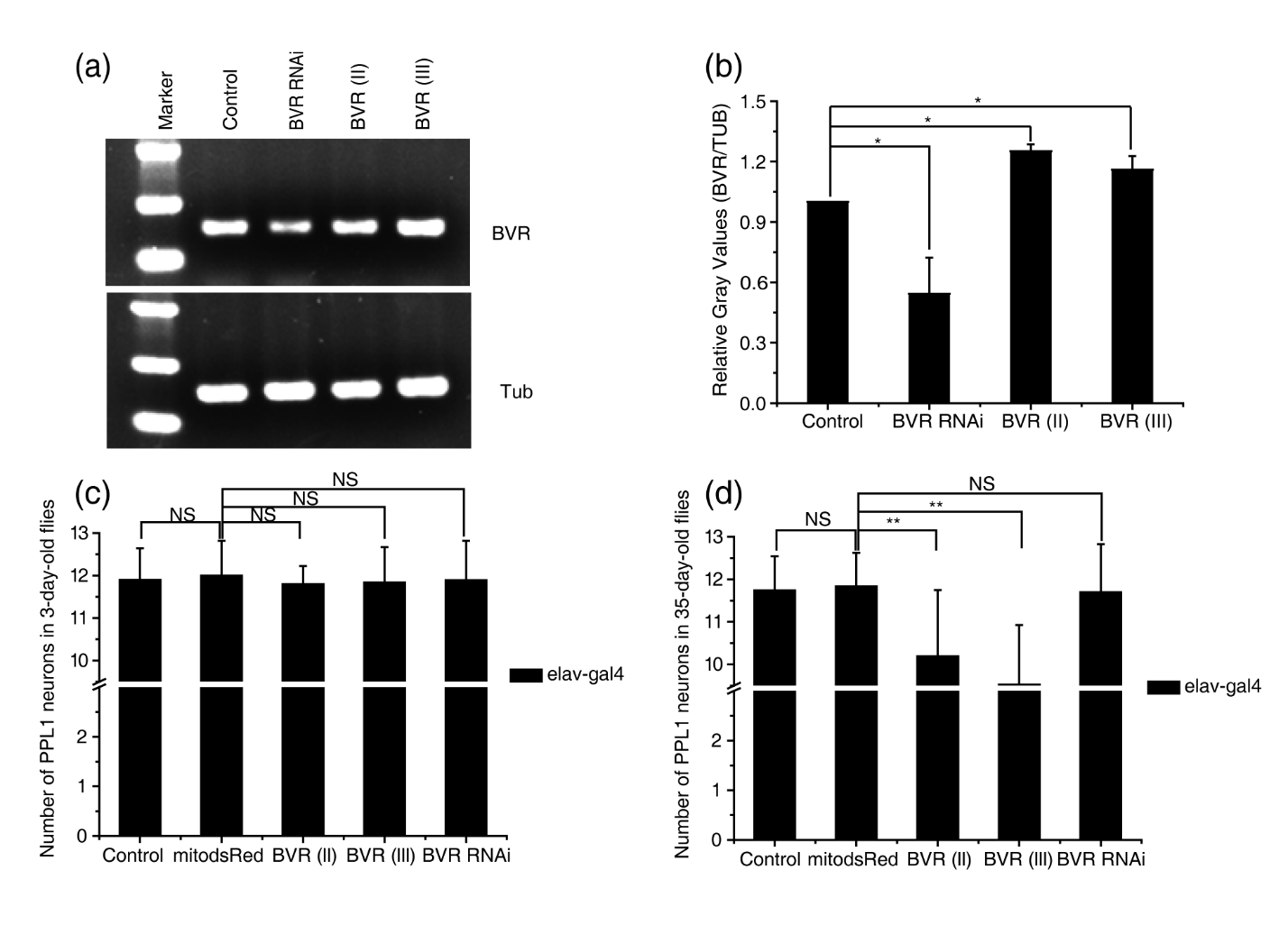


**Supplementary Figure S3**


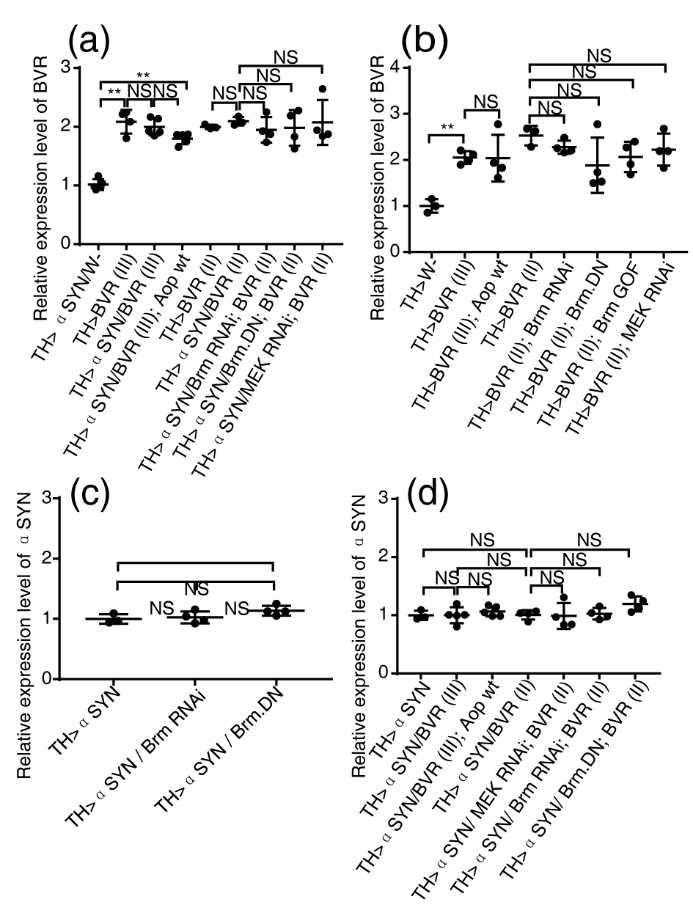


**Supplementary Figure S4**


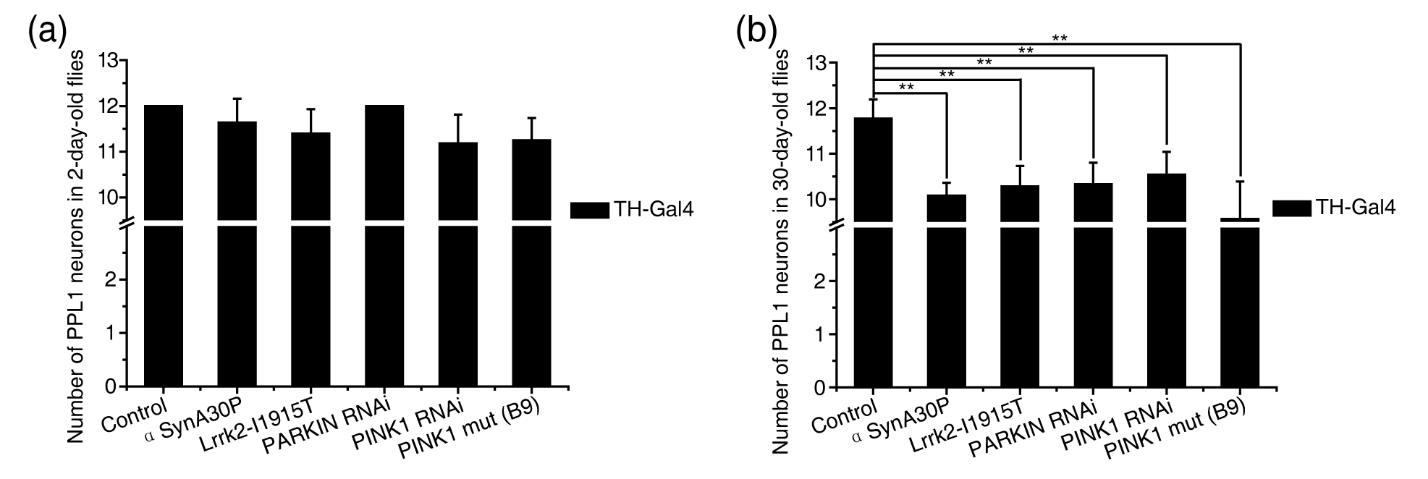


**Supplementary Figure S5**


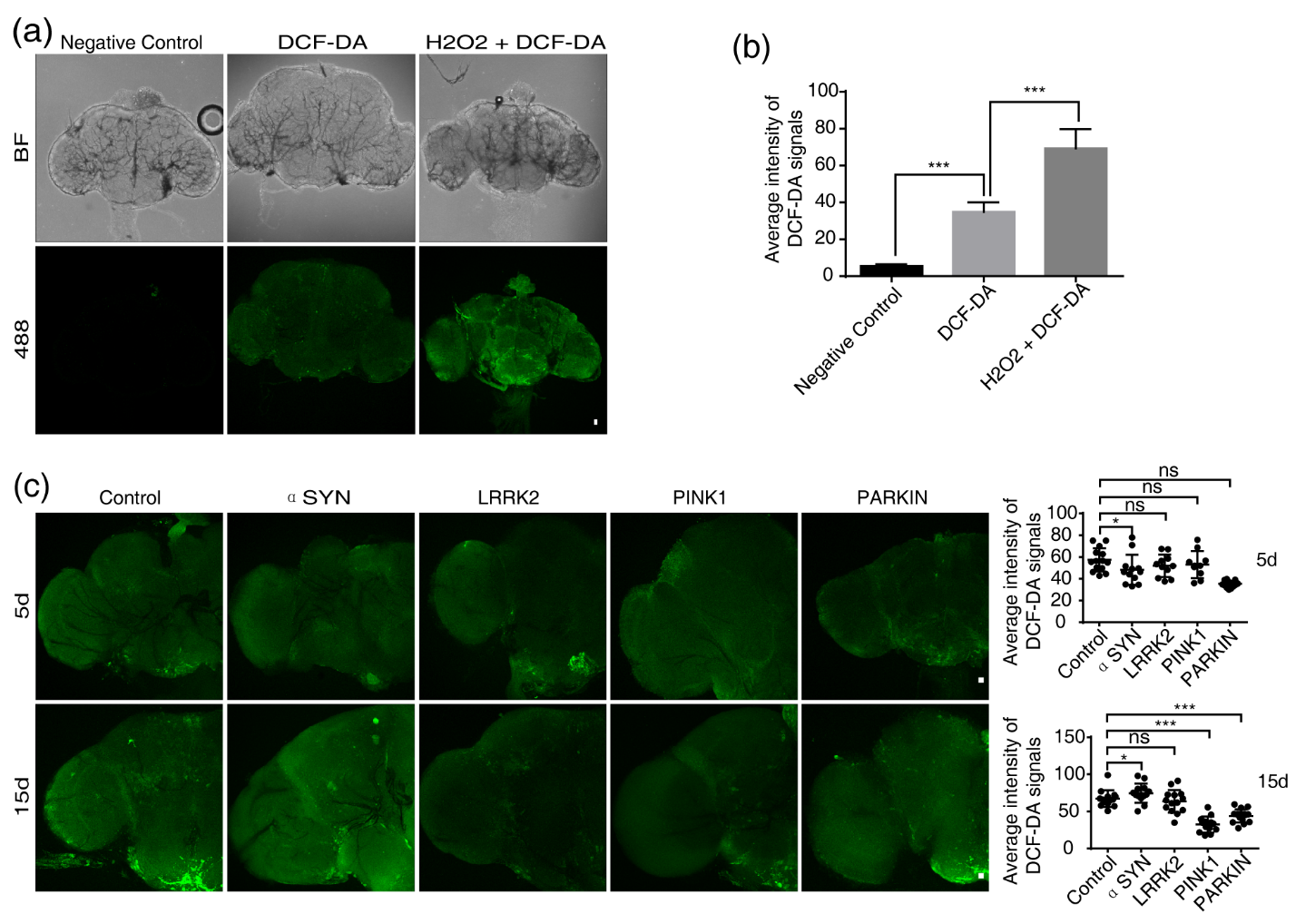


**Supplementary Figure S6**


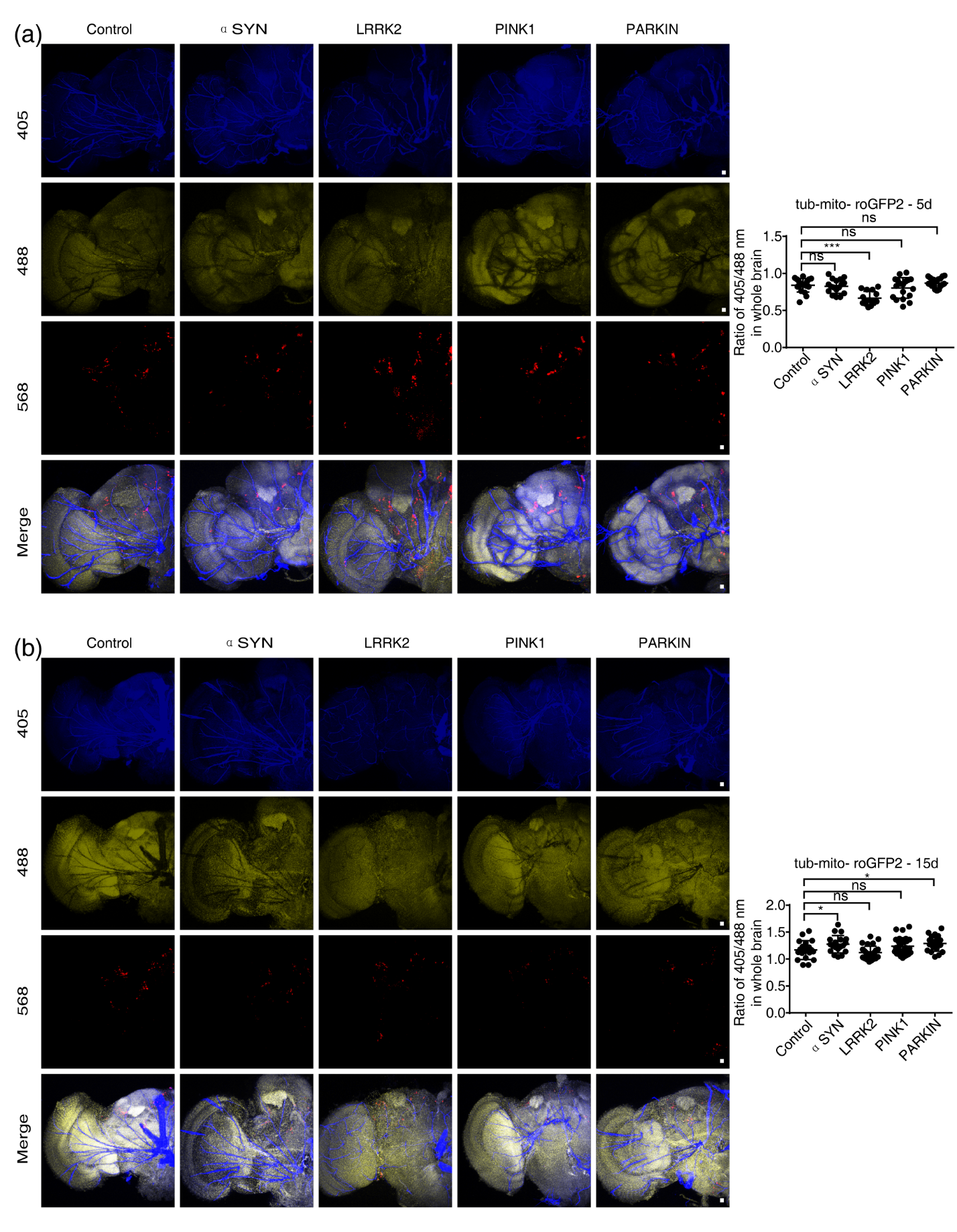


**Supplementary Figure S7**


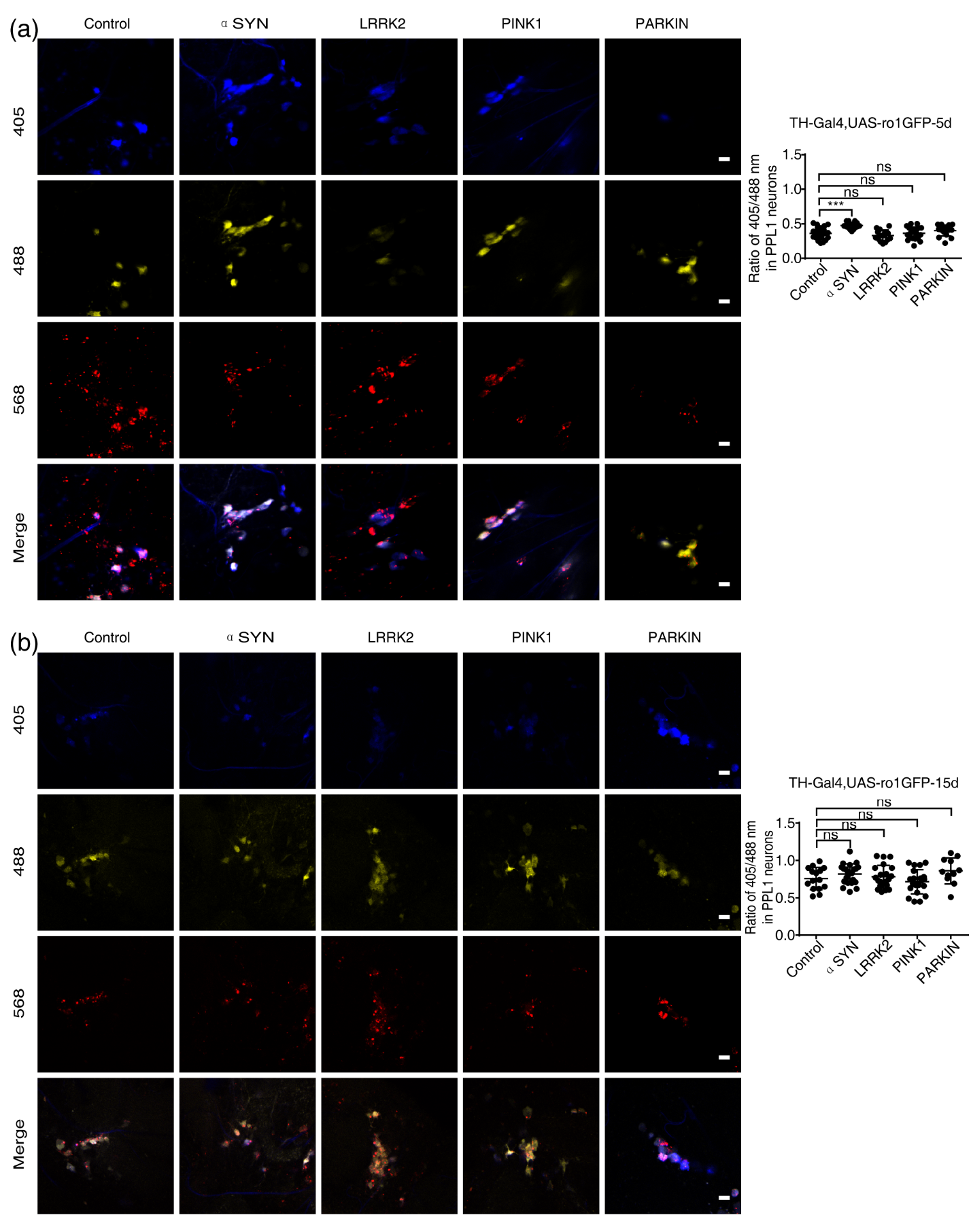


**Supplementary Figure S8**


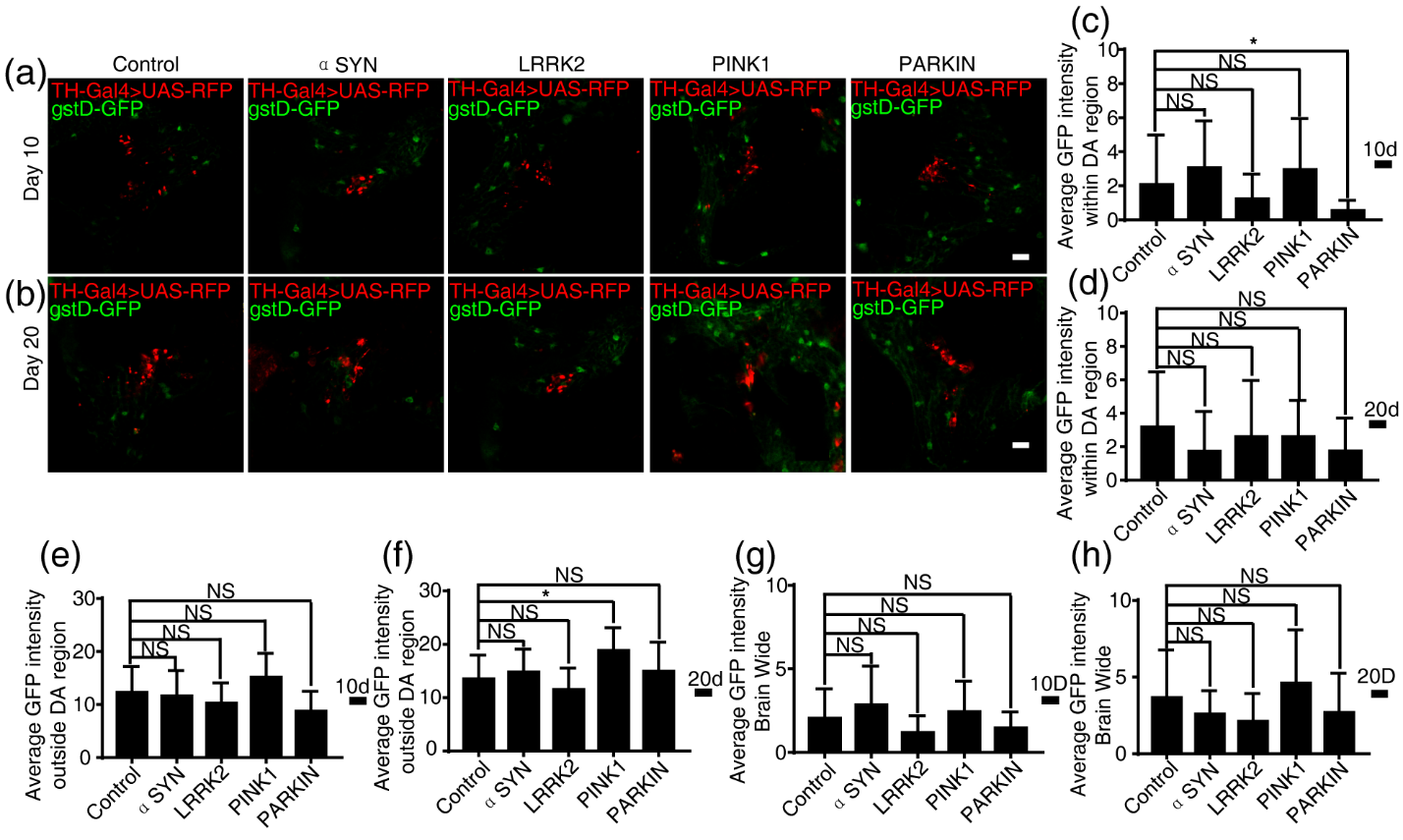


**Supplementary Figure S9**


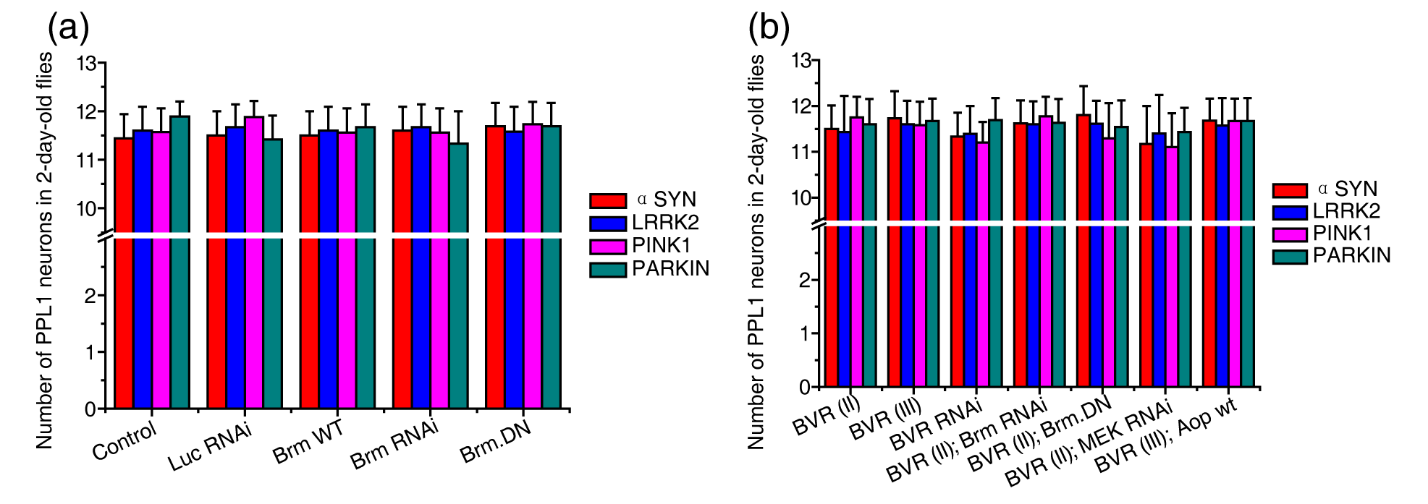


**Supplementary Figure S10**


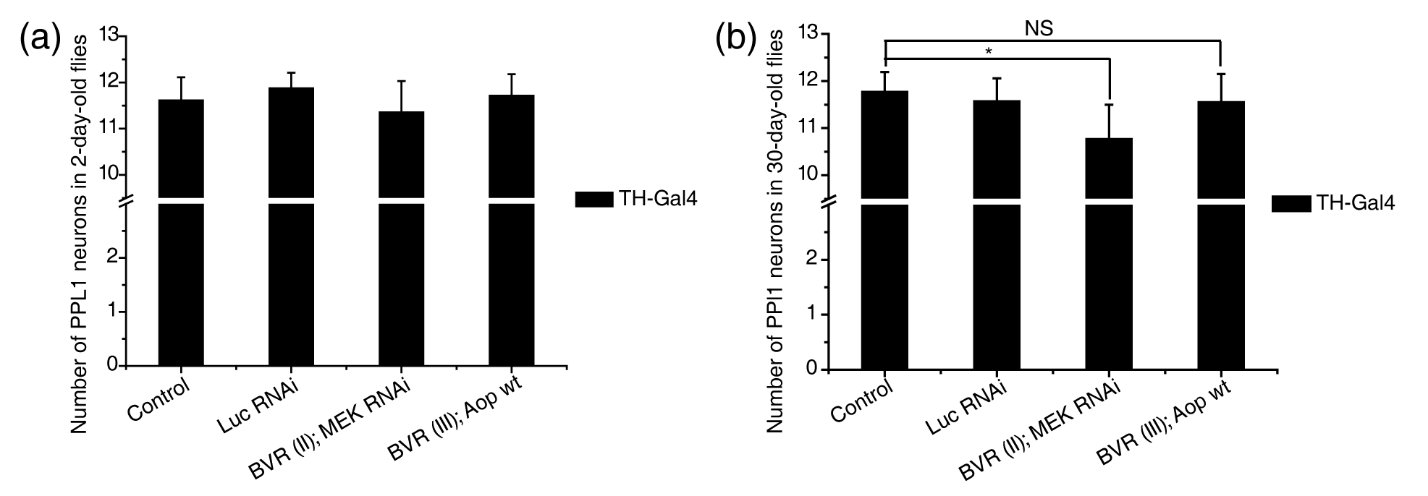


**Supplementary Figure S11**
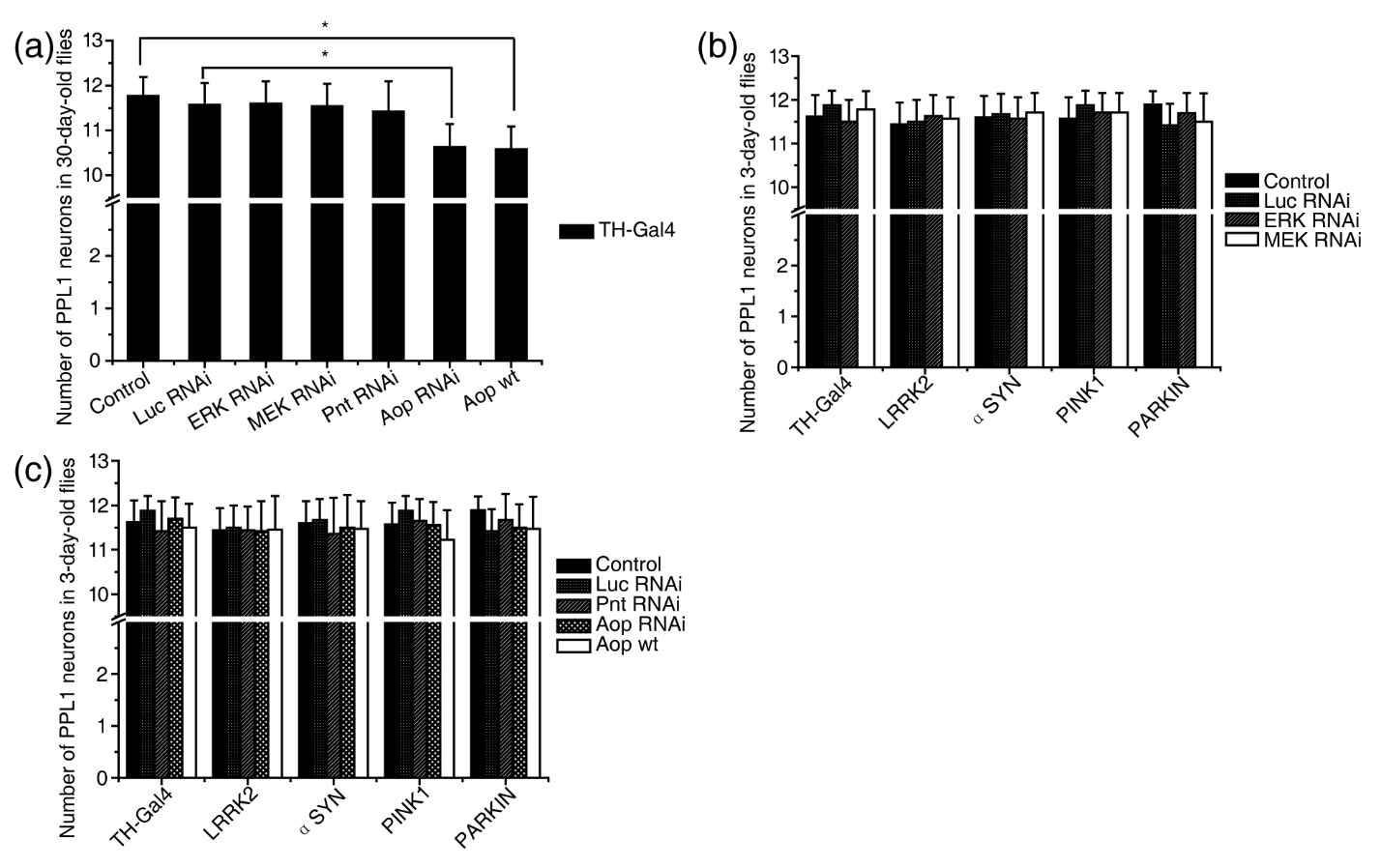


**Supplementary Figure S12**


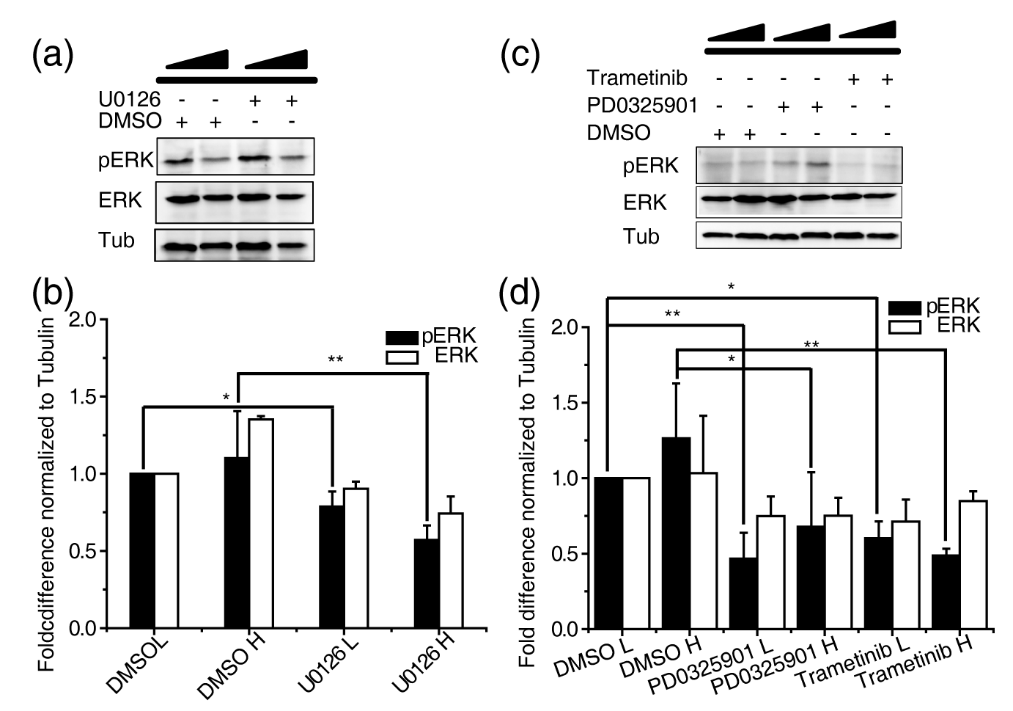


**Supplementary Table S1. Correlation of candidate genes with anchor genes**

| **Relativity** | **ATP13A2** | **SCNA** | **HTRA2** | **PARK2** | **LRRK2** | **PARK7** | **PINK1** |
| --- | --- | --- | --- | --- | --- | --- | --- |
| SMARCA4 |  | ※ | ※ |  | ※ | ※ | ※ |
| UBE3A |  | ※ |  |  | ※ | ※ | ※ |
| SNRPN |  | ※ |  |  | ※ | ※ | ※ |
| SLC25A3 |  | ※ | ※ |  |  | ※ | ※ |
| PRDX2 |  | ※ | ※ |  |  | ※ | ※ |
| GNAS | ※ | ※ | ※ |  |  |  | ※ |
| ARF1 | ※ | ※ | ※ |  |  | ※ |  |
| ACTG1 |  | ※ | ※ |  |  | ※ | ※ |
| VCP |  | ※ |  |  |  | ※ | ※ |
| TTC3 |  | ※ |  |  |  | ※ | ※ |
| STMN2 | ※ | ※ |  |  |  |  | ※ |
| RNF187 | ※ | ※ |  |  |  |  | ※ |
| OBSL1 | ※ | ※ |  |  |  |  | ※ |
| OAZ1 |  | ※ |  |  |  | ※ | ※ |
| NSFL1C | ※ | ※ |  |  |  | ※ |  |
| NTRK3 |  |  |  |  | ※ | ※ | ※ |
| MAP2K4 | ※ | ※ |  |  |  |  | ※ |
| LARP1 |  | ※ |  |  |  | ※ | ※ |
| KLC1 | ※ |  |  |  |  | ※ | ※ |
| IGHG1 | ※ |  | ※ |  | ※ |  |  |
| GAPDH |  |  | ※ |  |  | ※ | ※ |
| GAP43 | ※ | ※ |  |  |  |  | ※ |
| EPB41L1 | ※ | ※ |  |  |  |  | ※ |
| DSTN |  | ※ |  |  |  | ※ | ※ |
| DKK3 |  | ※ |  |  |  | ※ | ※ |
| CLTA | ※ |  | ※ |  |  | ※ |  |
| BLVRA | ※ | ※ |  |  |  |  | ※ |
| ATP6V1C1 | ※ | ※ |  |  |  | ※ |  |
| ATP50 |  | ※ | ※ |  |  | ※ |  |
| ARF3 |  | ※ |  |  |  | ※ | ※ |
| AAK1 |  | ※ |  |  | ※ | ※ |  |

Note: ※ represents high correlation between the candidate and the anchor gene.

**Supplementary Table S2. Pearson correlation coefficients (PCC ) value between anchors genes and SMARCA4/BLVRA**

| HTRA2 | **GDS2519** | **GDS2821** | **GDS3128** | **GSE19587** | **GSE20141** | **GSE20146** | **GSE20153** | **GSE20295** | **GSE20333** | **GDS3129** | **Median** |
| --- | --- | --- | --- | --- | --- | --- | --- | --- | --- | --- | --- |
| SMARCA4 | -0.134385 | 0.401202 | 0.434356 | -0.33275 | 0.778465 | 0.443811 | 0.544739 | 0.940379 | -0.380482 |  | 0.434356 |
| BLVRA | 0.130024 | 0.305576 | 0.514849 | 0.452213 | 0.259953 | 0.350115 | -0.427627 | 0.914044 | -0.267632 |  | 0.305576 |
| LRRK2 |  |  |  |  |  |  |  |  |  |  |  |
| SMARCA4 |  | 0.464852 |  | NA | -0.578301 | -0.4052 | 0.412829 | NA | NA | NA | 0.0038145 |
| BLVRA |  | 0.354546 |  | NA | -0.598422 | 0.406208 | 0.418558 | NA | NA | -0.113976 | 0.354546 |
| PARK2 |  |  |  |  |  |  |  |  |  |  |  |
| SMARCA4 | -0.181832 | 0.448349 | 0.759123 | 0.777099 | 0.217334 | 0.743286 | -0.38967 | 0.952408 | -0.458491 |  | 0.448349 |
| BLVRA | 0.226116 | -0.22987 | 0.73218 | 0.581159 | 0.233454 | -0.531026 | 0.32598 | 0.747416 | -0.077776 |  | 0.233454 |
| PARK7 |  |  |  |  |  |  |  |  |  |  |  |
| SMARCA4 | 0.399054 | 0.653309 |  | 0.696794 | 0.653024 | 0.462393 | 0.387262 | 0.976791 | -0.131253 | NA | 0.5577085 |
| BLVRA | 0.248587 | 0.730552 |  | -0.644057 | 0.811627 | 0.266754 | 0.265855 | 0.960803 | 0.379182 | -0.157136 | 0.266754 |
| PINK1 |  |  |  |  |  |  |  |  |  |  |  |
| SMARCA4 | 0.19246 | 0.608031 | 0.838268 | 0.716564 | 0.9534 | 0.684522 | -0.508087 | 0.932782 | NA |  | 0.700543 |
| BLVRA | -0.292087 | 0.569467 | 0.672501 | -0.574555 | 0.865426 | 0.362951 | -0.489034 | 0.558772 | NA |  | 0.4608615 |
| SNCA |  |  |  |  |  |  |  |  |  |  |  |
| SMARCA4 | -0.285349 | 0.689499 | 0.650606 | 0.698502 | 0.786245 | 0.524943 | 0.426371 | 0.950556 | 0.200241 |  | 0.650606 |
| BLVRA | -0.271254 | 0.525958 | 0.685866 | -0.378715 | 0.724884 | -0.552131 | 0.321484 | 0.924258 | 0.458517 | -0.528703 | 0.458517 |

Note: HTRA2, LRRK2, PARK2, PARK7, PINK1 and SNCA are anchor genes; GDS2519, GDS2821, GDS3128, GSE19587, GSE20141, GSE20146, GSE20153, GSE20295, GSE20333 and GDS3129 are datasets from NCBI Gene Expression Omnibus (GEO). Top 5% PCC are highlighted in yellow.

**Supplementary Table S3. SMARCA4 PD risk SNPs**

| **[Polymorphism](http://www.pdgene.org/view?gene=SMARCA4)** | **[Location (hg19)](http://www.pdgene.org/view?gene=SMARCA4)** | **Gene** | **[Ethnicity](http://www.pdgene.org/view?gene=SMARCA4)** | **[# Samples](http://www.pdgene.org/view?gene=SMARCA4)** | **[# Studies](http://www.pdgene.org/view?gene=SMARCA4)** | **[Allele contrast](http://www.pdgene.org/view?gene=SMARCA4)** | **1000G CEU** | **1000G CHB+JPT** | **Meta OR (95%CI)** | **I2 (95%CI)** | **[Meta](http://www.pdgene.org/view?gene=SMARCA4)**  [**P-value**](http://www.pdgene.org/view?gene=SMARCA4) |
| --- | --- | --- | --- | --- | --- | --- | --- | --- | --- | --- | --- |
| [rs9105](http://www.pdgene.org/view?poly=rs9105) | [chr19:11169514](http://genome.ucsc.edu/cgi-bin/hgTracks?org=human&hgt.customText=http://www.pdgene.org/tracks?hg=hg19&db=hg19&position=chr19%3A11169264-11169764&hgt.suggest=&pix=800&Submit=submit&hgsid=184845147) | [SMARCA4](http://www.pdgene.org/view?gene=SMARCA4) | C | - | 12 | T vs. C | 0.058 (T) | - | <1 (-) | 0 (-) | <0.05 |
| [rs150323051](http://www.pdgene.org/view?poly=rs150323051) | [chr19:11058880](http://genome.ucsc.edu/cgi-bin/hgTracks?org=human&hgt.customText=http://www.pdgene.org/tracks?hg=hg19&db=hg19&position=chr19%3A11058630-11059130&hgt.suggest=&pix=800&Submit=submit&hgsid=184845147) | [SMARCA4[-12718bp]](http://www.pdgene.org/view?gene=SMARCA4) | C | - | 11 | A vs. G | - | - | <1 (-) | 0 (-) | <0.05 |
| [rs117234045](http://www.pdgene.org/view?poly=rs117234045) | [chr19:11127139](http://genome.ucsc.edu/cgi-bin/hgTracks?org=human&hgt.customText=http://www.pdgene.org/tracks?hg=hg19&db=hg19&position=chr19%3A11126889-11127389&hgt.suggest=&pix=800&Submit=submit&hgsid=184845147) | [SMARCA4](http://www.pdgene.org/view?gene=SMARCA4) | C | - | 11 | A vs. G | 0.008 (A) | - | >1 (-) | 0 (-) | <0.05 |
| [rs118107587](http://www.pdgene.org/view?poly=rs118107587) | [chr19:11134752](http://genome.ucsc.edu/cgi-bin/hgTracks?org=human&hgt.customText=http://www.pdgene.org/tracks?hg=hg19&db=hg19&position=chr19%3A11134502-11135002&hgt.suggest=&pix=800&Submit=submit&hgsid=184845147) | [SMARCA4](http://www.pdgene.org/view?gene=SMARCA4) | C | - | 11 | A vs. G | 0.05 (A) | - | <1 (-) | 0 (-) | <0.05 |
| [rs117819913](http://www.pdgene.org/view?poly=rs117819913) | [chr19:11123091](http://genome.ucsc.edu/cgi-bin/hgTracks?org=human&hgt.customText=http://www.pdgene.org/tracks?hg=hg19&db=hg19&position=chr19%3A11122841-11123341&hgt.suggest=&pix=800&Submit=submit&hgsid=184845147) | [SMARCA4](http://www.pdgene.org/view?gene=SMARCA4) | C | - | 10 | A vs. T | 0.05 (A) | - | >1 (-) | 0 (-) | <0.05 |
| [rs12609500](http://www.pdgene.org/view?poly=rs12609500) | [chr19:11173928](http://genome.ucsc.edu/cgi-bin/hgTracks?org=human&hgt.customText=http://www.pdgene.org/tracks?hg=hg19&db=hg19&position=chr19%3A11173678-11174178&hgt.suggest=&pix=800&Submit=submit&hgsid=184845147) | [SMARCA4](http://www.pdgene.org/view?gene=SMARCA4) | C | - | 12 | T vs. C | - | - | <1 (-) | 0 (-) | <0.05 |
| [rs73013198](http://www.pdgene.org/view?poly=rs73013198) | [chr19:11174742](http://genome.ucsc.edu/cgi-bin/hgTracks?org=human&hgt.customText=http://www.pdgene.org/tracks?hg=hg19&db=hg19&position=chr19%3A11174492-11174992&hgt.suggest=&pix=800&Submit=submit&hgsid=184845147) | [SMARCA4](http://www.pdgene.org/view?gene=SMARCA4) | C | - | 12 | T vs. C | 0.242 (T) | 0.05 (T) | <1 (-) | 0 (-) | <0.05 |
| [rs73013202](http://www.pdgene.org/view?poly=rs73013202) | [chr19:11179709](http://genome.ucsc.edu/cgi-bin/hgTracks?org=human&hgt.customText=http://www.pdgene.org/tracks?hg=hg19&db=hg19&position=chr19%3A11179459-11179959&hgt.suggest=&pix=800&Submit=submit&hgsid=184845147) | [SMARCA4[+3638bp]](http://www.pdgene.org/view?gene=SMARCA4) | C | - | 12 | C vs. G | 0.25 (G) | 0.067 (G) | >1 (-) | 0 (-) | <0.05 |
| [rs141989097](http://www.pdgene.org/view?poly=rs141989097) | [chr19:11069069](http://genome.ucsc.edu/cgi-bin/hgTracks?org=human&hgt.customText=http://www.pdgene.org/tracks?hg=hg19&db=hg19&position=chr19%3A11068819-11069319&hgt.suggest=&pix=800&Submit=submit&hgsid=184845147) | [SMARCA4[-2529bp]](http://www.pdgene.org/view?gene=SMARCA4) | C | - | 12 | T vs. C | - | - | >1 (-) | 23 (-) | <0.05 |
| [rs112186070](http://www.pdgene.org/view?poly=rs112186070) | [chr19:11168261](http://genome.ucsc.edu/cgi-bin/hgTracks?org=human&hgt.customText=http://www.pdgene.org/tracks?hg=hg19&db=hg19&position=chr19%3A11168011-11168511&hgt.suggest=&pix=800&Submit=submit&hgsid=184845147) | [SMARCA4](http://www.pdgene.org/view?gene=SMARCA4) | C | - | 12 | T vs. C | - | - | <1 (-) | 0 (-) | <0.05 |
| [rs113113862](http://www.pdgene.org/view?poly=rs113113862) | [chr19:11183577](http://genome.ucsc.edu/cgi-bin/hgTracks?org=human&hgt.customText=http://www.pdgene.org/tracks?hg=hg19&db=hg19&position=chr19%3A11183327-11183827&hgt.suggest=&pix=800&Submit=submit&hgsid=184845147) | [SMARCA4[+7506bp]](http://www.pdgene.org/view?gene=SMARCA4) | C | - | 12 | A vs. G | - | - | <1 (-) | 0 (-) | <0.05 |
| [rs73015007](http://www.pdgene.org/view?poly=rs73015007) | [chr19:11183837](http://genome.ucsc.edu/cgi-bin/hgTracks?org=human&hgt.customText=http://www.pdgene.org/tracks?hg=hg19&db=hg19&position=chr19%3A11183587-11184087&hgt.suggest=&pix=800&Submit=submit&hgsid=184845147) | [SMARCA4[+7766bp]](http://www.pdgene.org/view?gene=SMARCA4) | C | - | 12 | A vs. G | 0.25 (A) | - | <1 (-) | 0 (-) | <0.05 |
| [rs1122608](http://www.pdgene.org/view?poly=rs1122608) | [chr19:11163601](http://genome.ucsc.edu/cgi-bin/hgTracks?org=human&hgt.customText=http://www.pdgene.org/tracks?hg=hg19&db=hg19&position=chr19%3A11163351-11163851&hgt.suggest=&pix=800&Submit=submit&hgsid=184845147) | [SMARCA4](http://www.pdgene.org/view?gene=SMARCA4) | C | - | 12 | T vs. G | 0.25 (T) | 0.108 (T) | <1 (-) | 0 (-) | <0.05 |
| [rs3786728](http://www.pdgene.org/view?poly=rs3786728) | [chr19:11168038](http://genome.ucsc.edu/cgi-bin/hgTracks?org=human&hgt.customText=http://www.pdgene.org/tracks?hg=hg19&db=hg19&position=chr19%3A11167788-11168288&hgt.suggest=&pix=800&Submit=submit&hgsid=184845147) | [SMARCA4](http://www.pdgene.org/view?gene=SMARCA4) | C | - | 12 | A vs. G | 0.25 (G) | 0.067 (G) | >1 (-) | 0 (-) | <0.05 |
| [rs3786722](http://www.pdgene.org/view?poly=rs3786722) | [chr19:11161537](http://genome.ucsc.edu/cgi-bin/hgTracks?org=human&hgt.customText=http://www.pdgene.org/tracks?hg=hg19&db=hg19&position=chr19%3A11161287-11161787&hgt.suggest=&pix=800&Submit=submit&hgsid=184845147) | [SMARCA4](http://www.pdgene.org/view?gene=SMARCA4) | C | - | 12 | A vs. C | 0.25 (A) | 0.108 (A) | <1 (-) | 0 (-) | <0.05 |
| [rs112369586](http://www.pdgene.org/view?poly=rs112369586) | [chr19:11164460](http://genome.ucsc.edu/cgi-bin/hgTracks?org=human&hgt.customText=http://www.pdgene.org/tracks?hg=hg19&db=hg19&position=chr19%3A11164210-11164710&hgt.suggest=&pix=800&Submit=submit&hgsid=184845147) | [SMARCA4](http://www.pdgene.org/view?gene=SMARCA4) | C | - | 12 | A vs. G | 0.25 (A) | 0.092 (A) | <1 (-) | 0 (-) | <0.05 |
| [rs12609863](http://www.pdgene.org/view?poly=rs12609863) | [chr19:11167219](http://genome.ucsc.edu/cgi-bin/hgTracks?org=human&hgt.customText=http://www.pdgene.org/tracks?hg=hg19&db=hg19&position=chr19%3A11166969-11167469&hgt.suggest=&pix=800&Submit=submit&hgsid=184845147) | [SMARCA4](http://www.pdgene.org/view?gene=SMARCA4) | C | - | 12 | T vs. C | 0.258 (T) | 0.108 (T) | <1 (-) | 0 (-) | <0.05 |
| [rs117210556](http://www.pdgene.org/view?poly=rs117210556) | [chr19:11126197](http://genome.ucsc.edu/cgi-bin/hgTracks?org=human&hgt.customText=http://www.pdgene.org/tracks?hg=hg19&db=hg19&position=chr19%3A11125947-11126447&hgt.suggest=&pix=800&Submit=submit&hgsid=184845147) | [SMARCA4](http://www.pdgene.org/view?gene=SMARCA4) | C | - | 9 | T vs. C | 0.058 (C) | - | <1 (-) | 0 (-) | <0.05 |
| [rs55948246](http://www.pdgene.org/view?poly=rs55948246) | [chr19:11159889](http://genome.ucsc.edu/cgi-bin/hgTracks?org=human&hgt.customText=http://www.pdgene.org/tracks?hg=hg19&db=hg19&position=chr19%3A11159639-11160139&hgt.suggest=&pix=800&Submit=submit&hgsid=184845147) | [SMARCA4](http://www.pdgene.org/view?gene=SMARCA4) | C | - | 12 | T vs. C | 0.25 (C) | 0.092 (C) | >1 (-) | 0 (-) | <0.05 |
| [rs12052058](http://www.pdgene.org/view?poly=rs12052058) | [chr19:11159525](http://genome.ucsc.edu/cgi-bin/hgTracks?org=human&hgt.customText=http://www.pdgene.org/tracks?hg=hg19&db=hg19&position=chr19%3A11159275-11159775&hgt.suggest=&pix=800&Submit=submit&hgsid=184845147) | [SMARCA4](http://www.pdgene.org/view?gene=SMARCA4) | C | - | 12 | T vs. G | 0.25 (T) | 0.1 (T) | <1 (-) | 0 (-) | <0.05 |
| [rs12052201](http://www.pdgene.org/view?poly=rs12052201) | [chr19:11159096](http://genome.ucsc.edu/cgi-bin/hgTracks?org=human&hgt.customText=http://www.pdgene.org/tracks?hg=hg19&db=hg19&position=chr19%3A11158846-11159346&hgt.suggest=&pix=800&Submit=submit&hgsid=184845147) | [SMARCA4](http://www.pdgene.org/view?gene=SMARCA4) | C | - | 12 | T vs. G | 0.25 (T) | 0.1 (T) | <1 (-) | 0 (-) | <0.05 |
| [rs79227604](http://www.pdgene.org/view?poly=rs79227604) | [chr19:11065183](http://genome.ucsc.edu/cgi-bin/hgTracks?org=human&hgt.customText=http://www.pdgene.org/tracks?hg=hg19&db=hg19&position=chr19%3A11064933-11065433&hgt.suggest=&pix=800&Submit=submit&hgsid=184845147) | [SMARCA4[-6415bp]](http://www.pdgene.org/view?gene=SMARCA4) | C | - | 12 | T vs. C | 0.1 (T) | - | >1 (-) | 0 (-) | <0.05 |
| [rs12052200](http://www.pdgene.org/view?poly=rs12052200) | [chr19:11159076](http://genome.ucsc.edu/cgi-bin/hgTracks?org=human&hgt.customText=http://www.pdgene.org/tracks?hg=hg19&db=hg19&position=chr19%3A11158826-11159326&hgt.suggest=&pix=800&Submit=submit&hgsid=184845147) | [SMARCA4](http://www.pdgene.org/view?gene=SMARCA4) | C | - | 12 | A vs. G | 0.325 (A) | 0.308 (A) | <1 (-) | 0 (-) | <0.05 |
| [rs76614166](http://www.pdgene.org/view?poly=rs76614166) | [chr19:11104243](http://genome.ucsc.edu/cgi-bin/hgTracks?org=human&hgt.customText=http://www.pdgene.org/tracks?hg=hg19&db=hg19&position=chr19%3A11103993-11104493&hgt.suggest=&pix=800&Submit=submit&hgsid=184845147) | [SMARCA4](http://www.pdgene.org/view?gene=SMARCA4) | C | - | 11 | A vs. G | 0.05 (G) | - | <1 (-) | 3 (-) | <0.05 |
| [rs118158676](http://www.pdgene.org/view?poly=rs118158676) | [chr19:11079212](http://genome.ucsc.edu/cgi-bin/hgTracks?org=human&hgt.customText=http://www.pdgene.org/tracks?hg=hg19&db=hg19&position=chr19%3A11078962-11079462&hgt.suggest=&pix=800&Submit=submit&hgsid=184845147) | [SMARCA4](http://www.pdgene.org/view?gene=SMARCA4) | C | - | 11 | T vs. C | 0.025 (T) | - | <1 (-) | 0 (-) | <0.05 |
| [rs3786721](http://www.pdgene.org/view?poly=rs3786721) | [chr19:11146499](http://genome.ucsc.edu/cgi-bin/hgTracks?org=human&hgt.customText=http://www.pdgene.org/tracks?hg=hg19&db=hg19&position=chr19%3A11146249-11146749&hgt.suggest=&pix=800&Submit=submit&hgsid=184845147) | [SMARCA4](http://www.pdgene.org/view?gene=SMARCA4) | C | - | 12 | T vs. C | 0.45 (T) | 0.125 (T) | >1 (-) | 0 (-) | <0.05 |
| [rs10417443](http://www.pdgene.org/view?poly=rs10417443) | [chr19:11129429](http://genome.ucsc.edu/cgi-bin/hgTracks?org=human&hgt.customText=http://www.pdgene.org/tracks?hg=hg19&db=hg19&position=chr19%3A11129179-11129679&hgt.suggest=&pix=800&Submit=submit&hgsid=184845147) | [SMARCA4](http://www.pdgene.org/view?gene=SMARCA4) | C | - | 12 | C vs. G | 0.483 (C) | 0.117 (C) | >1 (-) | 0 (-) | <0.05 |
| [rs34285220](http://www.pdgene.org/view?poly=rs34285220) | [chr19:11184260](http://genome.ucsc.edu/cgi-bin/hgTracks?org=human&hgt.customText=http://www.pdgene.org/tracks?hg=hg19&db=hg19&position=chr19%3A11184010-11184510&hgt.suggest=&pix=800&Submit=submit&hgsid=184845147) | [SMARCA4[+8189bp]](http://www.pdgene.org/view?gene=SMARCA4) | C | - | 11 | T vs. C | - | - | >1 (-) | 9 (-) | <0.05 |
| [rs78301016](http://www.pdgene.org/view?poly=rs78301016) | [chr19:11063046](http://genome.ucsc.edu/cgi-bin/hgTracks?org=human&hgt.customText=http://www.pdgene.org/tracks?hg=hg19&db=hg19&position=chr19%3A11062796-11063296&hgt.suggest=&pix=800&Submit=submit&hgsid=184845147) | [SMARCA4[-8552bp]](http://www.pdgene.org/view?gene=SMARCA4) | C | - | 12 | A vs. G | 0.183 (A) | 0.025 (A) | >1 (-) | 30 (-) | <0.05 |

**Supplementary Table S4. BLVRA PD risk SNPs**

| [**Polymorphism**](http://www.pdgene.org/view?gene=BLVRA) | [**Location (hg19)**](http://www.pdgene.org/view?gene=BLVRA) | **Gene** | [**Ethnicity**](http://www.pdgene.org/view?gene=BLVRA) | [**# Samples**](http://www.pdgene.org/view?gene=BLVRA) | [**# Studies**](http://www.pdgene.org/view?gene=BLVRA) | [**Allele contrast**](http://www.pdgene.org/view?gene=BLVRA) | **1000G CEU** | **1000G CHB+JPT** | **Meta OR (95%CI)** | **I2 (95%CI)** | [**Meta P-value**](http://www.pdgene.org/view?gene=BLVRA) |
| --- | --- | --- | --- | --- | --- | --- | --- | --- | --- | --- | --- |
| [rs1813599](http://www.pdgene.org/view?poly=rs1813599) | [chr7:43869788](http://genome.ucsc.edu/cgi-bin/hgTracks?org=human&hgt.customText=http://www.pdgene.org/tracks?hg=hg19&db=hg19&position=chr7%3A43869538-43870038&hgt.suggest=&pix=800&Submit=submit&hgsid=184845147) | [BLVRA[+22849bp]](http://www.pdgene.org/view?gene=BLVRA) | C | - | 13 | A vs. G | - | - | >1 (-) | 6 (-) | <0.05 |
| [rs10951743](http://www.pdgene.org/view?poly=rs10951743) | [chr7:43870113](http://genome.ucsc.edu/cgi-bin/hgTracks?org=human&hgt.customText=http://www.pdgene.org/tracks?hg=hg19&db=hg19&position=chr7%3A43869863-43870363&hgt.suggest=&pix=800&Submit=submit&hgsid=184845147) | [BLVRA[+23174bp]](http://www.pdgene.org/view?gene=BLVRA) | C | - | 13 | T vs. C | 0.383 (C) | - | <1 (-) | 0 (-) | <0.05 |
| [rs6978907](http://www.pdgene.org/view?poly=rs6978907) | [chr7:43835607](http://genome.ucsc.edu/cgi-bin/hgTracks?org=human&hgt.customText=http://www.pdgene.org/tracks?hg=hg19&db=hg19&position=chr7%3A43835357-43835857&hgt.suggest=&pix=800&Submit=submit&hgsid=184845147) | [BLVRA](http://www.pdgene.org/view?gene=BLVRA) | C | - | 13 | A vs. G | - | - | <1 (-) | 0 (-) | <0.05 |
| [rs146815437](http://www.pdgene.org/view?poly=rs146815437) | [chr7:43829380](http://genome.ucsc.edu/cgi-bin/hgTracks?org=human&hgt.customText=http://www.pdgene.org/tracks?hg=hg19&db=hg19&position=chr7%3A43829130-43829630&hgt.suggest=&pix=800&Submit=submit&hgsid=184845147) | [BLVRA](http://www.pdgene.org/view?gene=BLVRA) | C | - | 10 | A vs. C | - | - | <1 (-) | 43 (-) | <0.05 |
| [rs6945433](http://www.pdgene.org/view?poly=rs6945433) | [chr7:43858926](http://genome.ucsc.edu/cgi-bin/hgTracks?org=human&hgt.customText=http://www.pdgene.org/tracks?hg=hg19&db=hg19&position=chr7%3A43858676-43859176&hgt.suggest=&pix=800&Submit=submit&hgsid=184845147) | [BLVRA[+11987bp]](http://www.pdgene.org/view?gene=BLVRA) | C | - | 13 | T vs. C | 0.333 (T) | 0.325 (T) | >1 (-) | 0 (-) | <0.05 |
| [rs143530356](http://www.pdgene.org/view?poly=rs143530356) | [chr7:43829579](http://genome.ucsc.edu/cgi-bin/hgTracks?org=human&hgt.customText=http://www.pdgene.org/tracks?hg=hg19&db=hg19&position=chr7%3A43829329-43829829&hgt.suggest=&pix=800&Submit=submit&hgsid=184845147) | [BLVRA](http://www.pdgene.org/view?gene=BLVRA) | C | - | 4 | C vs. G | - | - | >1 (-) | 0 (-) | <0.05 |
| [rs3094952](http://www.pdgene.org/view?poly=rs3094952) | [chr7:43837562](http://genome.ucsc.edu/cgi-bin/hgTracks?org=human&hgt.customText=http://www.pdgene.org/tracks?hg=hg19&db=hg19&position=chr7%3A43837312-43837812&hgt.suggest=&pix=800&Submit=submit&hgsid=184845147) | [BLVRA](http://www.pdgene.org/view?gene=BLVRA) | C | - | 13 | A vs. G | - | - | <1 (-) | 0 (-) | <0.05 |
| [rs34865291](http://www.pdgene.org/view?poly=rs34865291) | [chr7:43864079](http://genome.ucsc.edu/cgi-bin/hgTracks?org=human&hgt.customText=http://www.pdgene.org/tracks?hg=hg19&db=hg19&position=chr7%3A43863829-43864329&hgt.suggest=&pix=800&Submit=submit&hgsid=184845147) | [BLVRA[+17140bp]](http://www.pdgene.org/view?gene=BLVRA) | C | - | 13 | A vs. G | 0.025 (G) | - | <1 (-) | 0 (-) | <0.05 |
| [rs147976254](http://www.pdgene.org/view?poly=rs147976254) | [chr7:43866701](http://genome.ucsc.edu/cgi-bin/hgTracks?org=human&hgt.customText=http://www.pdgene.org/tracks?hg=hg19&db=hg19&position=chr7%3A43866451-43866951&hgt.suggest=&pix=800&Submit=submit&hgsid=184845147) | [BLVRA[+19762bp]](http://www.pdgene.org/view?gene=BLVRA) | C | - | 12 | A vs. G | - | - | >1 (-) | 0 (-) | <0.05 |
| [rs2282922](http://www.pdgene.org/view?poly=rs2282922) | [chr7:43844467](http://genome.ucsc.edu/cgi-bin/hgTracks?org=human&hgt.customText=http://www.pdgene.org/tracks?hg=hg19&db=hg19&position=chr7%3A43844217-43844717&hgt.suggest=&pix=800&Submit=submit&hgsid=184845147) | [BLVRA](http://www.pdgene.org/view?gene=BLVRA) | C | - | 13 | T vs. C | 0.283 (C) | 0.267 (C) | >1 (-) | 0 (-) | <0.05 |
| [rs1181602](http://www.pdgene.org/view?poly=rs1181602) | [chr7:43795677](http://genome.ucsc.edu/cgi-bin/hgTracks?org=human&hgt.customText=http://www.pdgene.org/tracks?hg=hg19&db=hg19&position=chr7%3A43795427-43795927&hgt.suggest=&pix=800&Submit=submit&hgsid=184845147) | [BLVRA[-2602bp]](http://www.pdgene.org/view?gene=BLVRA) | C | - | 13 | T vs. G | - | - | <1 (-) | 0 (-) | <0.05 |
| [rs2730625](http://www.pdgene.org/view?poly=rs2730625) | [chr7:43845944](http://genome.ucsc.edu/cgi-bin/hgTracks?org=human&hgt.customText=http://www.pdgene.org/tracks?hg=hg19&db=hg19&position=chr7%3A43845694-43846194&hgt.suggest=&pix=800&Submit=submit&hgsid=184845147) | [BLVRA](http://www.pdgene.org/view?gene=BLVRA) | C | - | 13 | T vs. C | 0.283 (C) | 0.267 (C) | >1 (-) | 0 (-) | <0.05 |
| [rs2299149](http://www.pdgene.org/view?poly=rs2299149) | [chr7:43845185](http://genome.ucsc.edu/cgi-bin/hgTracks?org=human&hgt.customText=http://www.pdgene.org/tracks?hg=hg19&db=hg19&position=chr7%3A43844935-43845435&hgt.suggest=&pix=800&Submit=submit&hgsid=184845147) | [BLVRA](http://www.pdgene.org/view?gene=BLVRA) | C | - | 13 | A vs. G | 0.283 (G) | 0.267 (G) | >1 (-) | 0 (-) | <0.05 |
| [rs849162](http://www.pdgene.org/view?poly=rs849162) | [chr7:43821852](http://genome.ucsc.edu/cgi-bin/hgTracks?org=human&hgt.customText=http://www.pdgene.org/tracks?hg=hg19&db=hg19&position=chr7%3A43821602-43822102&hgt.suggest=&pix=800&Submit=submit&hgsid=184845147) | [BLVRA](http://www.pdgene.org/view?gene=BLVRA) | C | - | 13 | A vs. G | 0.283 (A) | 0.267 (A) | <1 (-) | 0 (-) | <0.05 |
| [rs699512](http://www.pdgene.org/view?poly=rs699512) | [chr7:43810764](http://genome.ucsc.edu/cgi-bin/hgTracks?org=human&hgt.customText=http://www.pdgene.org/tracks?hg=hg19&db=hg19&position=chr7%3A43810514-43811014&hgt.suggest=&pix=800&Submit=submit&hgsid=184845147) | [BLVRA](http://www.pdgene.org/view?gene=BLVRA) | C | - | 13 | A vs. G | 0.283 (G) | 0.267 (G) | >1 (-) | 0 (-) | <0.05 |
| [rs699510](http://www.pdgene.org/view?poly=rs699510) | [chr7:43810270](http://genome.ucsc.edu/cgi-bin/hgTracks?org=human&hgt.customText=http://www.pdgene.org/tracks?hg=hg19&db=hg19&position=chr7%3A43810020-43810520&hgt.suggest=&pix=800&Submit=submit&hgsid=184845147) | [BLVRA](http://www.pdgene.org/view?gene=BLVRA) | C | - | 13 | T vs. C | 0.283 (C) | 0.267 (C) | >1 (-) | 0 (-) | <0.05 |
| [rs2528369](http://www.pdgene.org/view?poly=rs2528369) | [chr7:43848123](http://genome.ucsc.edu/cgi-bin/hgTracks?org=human&hgt.customText=http://www.pdgene.org/tracks?hg=hg19&db=hg19&position=chr7%3A43847873-43848373&hgt.suggest=&pix=800&Submit=submit&hgsid=184845147) | [BLVRA[+1184bp]](http://www.pdgene.org/view?gene=BLVRA) | C | - | 13 | T vs. C | 0.267 (T) | 0.267 (T) | <1 (-) | 0 (-) | <0.05 |
| [rs2246171](http://www.pdgene.org/view?poly=rs2246171) | [chr7:43840347](http://genome.ucsc.edu/cgi-bin/hgTracks?org=human&hgt.customText=http://www.pdgene.org/tracks?hg=hg19&db=hg19&position=chr7%3A43840097-43840597&hgt.suggest=&pix=800&Submit=submit&hgsid=184845147) | [BLVRA](http://www.pdgene.org/view?gene=BLVRA) | C | - | 13 | A vs. G | 0.267 (G) | 0.267 (G) | >1 (-) | 0 (-) | <0.05 |
| [rs3094951](http://www.pdgene.org/view?poly=rs3094951) | [chr7:43840777](http://genome.ucsc.edu/cgi-bin/hgTracks?org=human&hgt.customText=http://www.pdgene.org/tracks?hg=hg19&db=hg19&position=chr7%3A43840527-43841027&hgt.suggest=&pix=800&Submit=submit&hgsid=184845147) | [BLVRA](http://www.pdgene.org/view?gene=BLVRA) | C | - | 13 | T vs. G | 0.267 (G) | 0.267 (G) | >1 (-) | 0 (-) | <0.05 |
| [rs1181573](http://www.pdgene.org/view?poly=rs1181573) | [chr7:43803607](http://genome.ucsc.edu/cgi-bin/hgTracks?org=human&hgt.customText=http://www.pdgene.org/tracks?hg=hg19&db=hg19&position=chr7%3A43803357-43803857&hgt.suggest=&pix=800&Submit=submit&hgsid=184845147) | [BLVRA](http://www.pdgene.org/view?gene=BLVRA) | C | - | 13 | A vs. G | 0.283 (G) | 0.267 (G) | >1 (-) | 0 (-) | <0.05 |
| [rs1317916](http://www.pdgene.org/view?poly=rs1317916) | [chr7:43839511](http://genome.ucsc.edu/cgi-bin/hgTracks?org=human&hgt.customText=http://www.pdgene.org/tracks?hg=hg19&db=hg19&position=chr7%3A43839261-43839761&hgt.suggest=&pix=800&Submit=submit&hgsid=184845147) | [BLVRA](http://www.pdgene.org/view?gene=BLVRA) | C | - | 13 | A vs. G | 0.267 (A) | 0.267 (A) | <1 (-) | 0 (-) | <0.05 |
| [rs1306743](http://www.pdgene.org/view?poly=rs1306743) | [chr7:43835218](http://genome.ucsc.edu/cgi-bin/hgTracks?org=human&hgt.customText=http://www.pdgene.org/tracks?hg=hg19&db=hg19&position=chr7%3A43834968-43835468&hgt.suggest=&pix=800&Submit=submit&hgsid=184845147) | [BLVRA](http://www.pdgene.org/view?gene=BLVRA) | C | - | 13 | A vs. T | 0.267 (T) | 0.267 (T) | >1 (-) | 0 (-) | <0.05 |
| [rs3107889](http://www.pdgene.org/view?poly=rs3107889) | [chr7:43838468](http://genome.ucsc.edu/cgi-bin/hgTracks?org=human&hgt.customText=http://www.pdgene.org/tracks?hg=hg19&db=hg19&position=chr7%3A43838218-43838718&hgt.suggest=&pix=800&Submit=submit&hgsid=184845147) | [BLVRA](http://www.pdgene.org/view?gene=BLVRA) | C | - | 13 | A vs. C | 0.275 (A) | 0.267 (A) | <1 (-) | 0 (-) | <0.05 |
| [rs1306742](http://www.pdgene.org/view?poly=rs1306742) | [chr7:43833162](http://genome.ucsc.edu/cgi-bin/hgTracks?org=human&hgt.customText=http://www.pdgene.org/tracks?hg=hg19&db=hg19&position=chr7%3A43832912-43833412&hgt.suggest=&pix=800&Submit=submit&hgsid=184845147) | [BLVRA](http://www.pdgene.org/view?gene=BLVRA) | C | - | 13 | T vs. C | 0.267 (T) | 0.267 (T) | <1 (-) | 0 (-) | <0.05 |
| [rs2730604](http://www.pdgene.org/view?poly=rs2730604) | [chr7:43850459](http://genome.ucsc.edu/cgi-bin/hgTracks?org=human&hgt.customText=http://www.pdgene.org/tracks?hg=hg19&db=hg19&position=chr7%3A43850209-43850709&hgt.suggest=&pix=800&Submit=submit&hgsid=184845147) | [BLVRA[+3520bp]](http://www.pdgene.org/view?gene=BLVRA) | C | - | 13 | A vs. C | 0.292 (C) | 0.283 (C) | >1 (-) | 0 (-) | <0.05 |
| [rs1637530](http://www.pdgene.org/view?poly=rs1637530) | [chr7:43797453](http://genome.ucsc.edu/cgi-bin/hgTracks?org=human&hgt.customText=http://www.pdgene.org/tracks?hg=hg19&db=hg19&position=chr7%3A43797203-43797703&hgt.suggest=&pix=800&Submit=submit&hgsid=184845147) | [BLVRA[-826bp]](http://www.pdgene.org/view?gene=BLVRA) | C | - | 13 | T vs. C | 0.283 (T) | 0.208 (T) | <1 (-) | 0 (-) | <0.05 |
| [rs3107888](http://www.pdgene.org/view?poly=rs3107888) | [chr7:43842117](http://genome.ucsc.edu/cgi-bin/hgTracks?org=human&hgt.customText=http://www.pdgene.org/tracks?hg=hg19&db=hg19&position=chr7%3A43841867-43842367&hgt.suggest=&pix=800&Submit=submit&hgsid=184845147) | [BLVRA](http://www.pdgene.org/view?gene=BLVRA) | C | - | 13 | A vs. T | 0.292 (T) | 0.267 (T) | >1 (-) | 0 (-) | <0.05 |
| [rs598042](http://www.pdgene.org/view?poly=rs598042) | [chr7:43873740](http://genome.ucsc.edu/cgi-bin/hgTracks?org=human&hgt.customText=http://www.pdgene.org/tracks?hg=hg19&db=hg19&position=chr7%3A43873490-43873990&hgt.suggest=&pix=800&Submit=submit&hgsid=184845147) | [BLVRA[+26801bp]](http://www.pdgene.org/view?gene=BLVRA) | C | - | 12 | A vs. C | 0.375 (C) | 0.417 (C) | >1 (-) | 0 (-) | <0.05 |
| [rs627932](http://www.pdgene.org/view?poly=rs627932) | [chr7:43871686](http://genome.ucsc.edu/cgi-bin/hgTracks?org=human&hgt.customText=http://www.pdgene.org/tracks?hg=hg19&db=hg19&position=chr7%3A43871436-43871936&hgt.suggest=&pix=800&Submit=submit&hgsid=184845147) | [BLVRA[+24747bp]](http://www.pdgene.org/view?gene=BLVRA) | C | - | 13 | A vs. C | 0.375 (C) | 0.417 (C) | >1 (-) | 0 (-) | <0.05 |
| [rs1181598](http://www.pdgene.org/view?poly=rs1181598) | [chr7:43789513](http://genome.ucsc.edu/cgi-bin/hgTracks?org=human&hgt.customText=http://www.pdgene.org/tracks?hg=hg19&db=hg19&position=chr7%3A43789263-43789763&hgt.suggest=&pix=800&Submit=submit&hgsid=184845147) | [BLVRA[-8766bp]](http://www.pdgene.org/view?gene=BLVRA) | C | - | 13 | C vs. G | 0.275 (C) | 0.267 (C) | <1 (-) | 0 (-) | <0.05 |
| [rs2529584](http://www.pdgene.org/view?poly=rs2529584) | [chr7:43790304](http://genome.ucsc.edu/cgi-bin/hgTracks?org=human&hgt.customText=http://www.pdgene.org/tracks?hg=hg19&db=hg19&position=chr7%3A43790054-43790554&hgt.suggest=&pix=800&Submit=submit&hgsid=184845147) | [BLVRA[-7975bp]](http://www.pdgene.org/view?gene=BLVRA) | C | - | 13 | A vs. G | 0.3 (A) | 0.267 (A) | <1 (-) | 0 (-) | <0.05 |
| [rs1181600](http://www.pdgene.org/view?poly=rs1181600) | [chr7:43789915](http://genome.ucsc.edu/cgi-bin/hgTracks?org=human&hgt.customText=http://www.pdgene.org/tracks?hg=hg19&db=hg19&position=chr7%3A43789665-43790165&hgt.suggest=&pix=800&Submit=submit&hgsid=184845147) | [BLVRA[-8364bp]](http://www.pdgene.org/view?gene=BLVRA) | C | - | 13 | T vs. C | 0.275 (T) | 0.267 (T) | <1 (-) | 0 (-) | <0.05 |
| [rs648048](http://www.pdgene.org/view?poly=rs648048) | [chr7:43861861](http://genome.ucsc.edu/cgi-bin/hgTracks?org=human&hgt.customText=http://www.pdgene.org/tracks?hg=hg19&db=hg19&position=chr7%3A43861611-43862111&hgt.suggest=&pix=800&Submit=submit&hgsid=184845147) | [BLVRA[+14922bp]](http://www.pdgene.org/view?gene=BLVRA) | C | - | 13 | A vs. G | - | - | <1 (-) | 0 (-) | <0.05 |
| [rs623108](http://www.pdgene.org/view?poly=rs623108) | [chr7:43864699](http://genome.ucsc.edu/cgi-bin/hgTracks?org=human&hgt.customText=http://www.pdgene.org/tracks?hg=hg19&db=hg19&position=chr7%3A43864449-43864949&hgt.suggest=&pix=800&Submit=submit&hgsid=184845147) | [BLVRA[+17760bp]](http://www.pdgene.org/view?gene=BLVRA) | C | - | 13 | A vs. G | 0.375 (A) | 0.417 (A) | <1 (-) | 0 (-) | <0.05 |
| [rs730585](http://www.pdgene.org/view?poly=rs730585) | [chr7:43789018](http://genome.ucsc.edu/cgi-bin/hgTracks?org=human&hgt.customText=http://www.pdgene.org/tracks?hg=hg19&db=hg19&position=chr7%3A43788768-43789268&hgt.suggest=&pix=800&Submit=submit&hgsid=184845147) | [BLVRA[-9261bp]](http://www.pdgene.org/view?gene=BLVRA) | C | - | 13 | A vs. G | 0.283 (A) | 0.267 (A) | <1 (-) | 0 (-) | <0.05 |
| [rs620833](http://www.pdgene.org/view?poly=rs620833) | [chr7:43865233](http://genome.ucsc.edu/cgi-bin/hgTracks?org=human&hgt.customText=http://www.pdgene.org/tracks?hg=hg19&db=hg19&position=chr7%3A43864983-43865483&hgt.suggest=&pix=800&Submit=submit&hgsid=184845147) | [BLVRA[+18294bp]](http://www.pdgene.org/view?gene=BLVRA) | C | - | 13 | C vs. G | 0.375 (G) | 0.417 (G) | >1 (-) | 0 (-) | <0.05 |
| [rs609979](http://www.pdgene.org/view?poly=rs609979) | [chr7:43873390](http://genome.ucsc.edu/cgi-bin/hgTracks?org=human&hgt.customText=http://www.pdgene.org/tracks?hg=hg19&db=hg19&position=chr7%3A43873140-43873640&hgt.suggest=&pix=800&Submit=submit&hgsid=184845147) | [BLVRA[+26451bp]](http://www.pdgene.org/view?gene=BLVRA) | C | - | 13 | A vs. C | 0.375 (A) | 0.417 (A) | <1 (-) | 0 (-) | <0.05 |
| [rs673402](http://www.pdgene.org/view?poly=rs673402) | [chr7:43873157](http://genome.ucsc.edu/cgi-bin/hgTracks?org=human&hgt.customText=http://www.pdgene.org/tracks?hg=hg19&db=hg19&position=chr7%3A43872907-43873407&hgt.suggest=&pix=800&Submit=submit&hgsid=184845147) | [BLVRA[+26218bp]](http://www.pdgene.org/view?gene=BLVRA) | C | - | 13 | A vs. G | - | - | <1 (-) | 0 (-) | <0.05 |
| [rs849180](http://www.pdgene.org/view?poly=rs849180) | [chr7:43785288](http://genome.ucsc.edu/cgi-bin/hgTracks?org=human&hgt.customText=http://www.pdgene.org/tracks?hg=hg19&db=hg19&position=chr7%3A43785038-43785538&hgt.suggest=&pix=800&Submit=submit&hgsid=184845147) | [BLVRA[-12991bp]](http://www.pdgene.org/view?gene=BLVRA) | C | - | 13 | T vs. C | - | - | >1 (-) | 0 (-) | <0.05 |
| [rs849179](http://www.pdgene.org/view?poly=rs849179) | [chr7:43785019](http://genome.ucsc.edu/cgi-bin/hgTracks?org=human&hgt.customText=http://www.pdgene.org/tracks?hg=hg19&db=hg19&position=chr7%3A43784769-43785269&hgt.suggest=&pix=800&Submit=submit&hgsid=184845147) | [BLVRA[-13260bp]](http://www.pdgene.org/view?gene=BLVRA) | C | - | 13 | A vs. T | 0.275 (T) | 0.267 (T) | >1 (-) | 0 (-) | <0.05 |
| [rs1181535](http://www.pdgene.org/view?poly=rs1181535) | [chr7:43874751](http://genome.ucsc.edu/cgi-bin/hgTracks?org=human&hgt.customText=http://www.pdgene.org/tracks?hg=hg19&db=hg19&position=chr7%3A43874501-43875001&hgt.suggest=&pix=800&Submit=submit&hgsid=184845147) | [BLVRA[+27812bp]](http://www.pdgene.org/view?gene=BLVRA) | C | - | 13 | A vs. G | 0.375 (G) | 0.417 (G) | >1 (-) | 0 (-) | <0.05 |
| [rs1181550](http://www.pdgene.org/view?poly=rs1181550) | [chr7:43861325](http://genome.ucsc.edu/cgi-bin/hgTracks?org=human&hgt.customText=http://www.pdgene.org/tracks?hg=hg19&db=hg19&position=chr7%3A43861075-43861575&hgt.suggest=&pix=800&Submit=submit&hgsid=184845147) | [BLVRA[+14386bp]](http://www.pdgene.org/view?gene=BLVRA) | C | - | 13 | A vs. G | 0.375 (G) | 0.417 (G) | >1 (-) | 0 (-) | <0.05 |
| [rs1181596](http://www.pdgene.org/view?poly=rs1181596) | [chr7:43785376](http://genome.ucsc.edu/cgi-bin/hgTracks?org=human&hgt.customText=http://www.pdgene.org/tracks?hg=hg19&db=hg19&position=chr7%3A43785126-43785626&hgt.suggest=&pix=800&Submit=submit&hgsid=184845147) | [BLVRA[-12903bp]](http://www.pdgene.org/view?gene=BLVRA) | C | - | 13 | A vs. G | - | - | <1 (-) | 0 (-) | <0.05 |
| [rs1181534](http://www.pdgene.org/view?poly=rs1181534) | [chr7:43875597](http://genome.ucsc.edu/cgi-bin/hgTracks?org=human&hgt.customText=http://www.pdgene.org/tracks?hg=hg19&db=hg19&position=chr7%3A43875347-43875847&hgt.suggest=&pix=800&Submit=submit&hgsid=184845147) | [BLVRA[+28658bp]](http://www.pdgene.org/view?gene=BLVRA) | C | - | 13 | A vs. G | 0.383 (G) | 0.417 (G) | >1 (-) | 0 (-) | <0.05 |

**Supplementary Table S6. Oligos used for the experiments**

| **gene** | **Forward Primer (5’→ 3’)** | **Reverse Primer (5’→ 3’)** |
| --- | --- | --- |
| αTUB | CGTTTGTCAAGCCTCATAGC | ACACCAGCCTGACCAACAT |
| BVR | ATAACGCGCTGGACATCCTC | TGCTATTATTGGAGGAACCGGC |
| αSYN | CCACAGTGGCTGAGAAGACC | AATTCCTTCCTGTGGGGCTC |

**APPENDIX: Fly genotype list**

**Figure 2**

Figure 2d, e

TH-Gal4/+

TH-Gal4; UAS-LucRNAi

TH-Gal4; UAS-Brm(wt)

TH-Gal4; UAS-Brm RNAi

TH-Gal4; UAS-Brm^DN^

TH-Gal4; UAS-Bvr II

TH-Gal4; UAS-Bvr III

TH-Gal4; UAS-Bvr RNAi

TH-Gal4; UAS-Bvr II; UAS-Brm^DN^

Figure 2f:

TH-Gal4; UAS-RFP/Brm::GFP

TH-Gal4, UAS-αSynA30P; UAS-RFP/Brm::GFP

TH-Gal4, UAS-RFP; UAS-dLrrk2^I1915T^/Brm::GFP

*pink1*^B9^; TH-Gal4, UAS-RFP; Brm::GFP

TH-Gal4, UAS-Parkin RNAi; UAS-RFP/Brm::GFP

**Figure 3**

Figure 3a, b:

TH-Gal4/+

TH-Gal4/UAS-Brm RNAi

TH-Gal4/UAS-Brm CA (GOF)

Figure 3c, d:

TH-Gal4, UAS-αSynA30P/+

TH-Gal4, UAS-αSynA30P; UAS-Brm RNAi

TH-Gal4, UAS-αSynA30P; UAS-Brm CA (GOF)

Figure 3e, f:

TH-Gal4; UAS- UAS-dLrrk2^I1915T^ /+

TH-Gal4; UAS-dLrrk2^I1915T^/UAS-Brm RNAi

TH-Gal4; UAS-dLrrk2^I1915T^/UAS-Brm CA (GOF)

**Figure 4**

Figure 4a:

TH-Gal4, UAS-αSynA30P/+

TH-Gal4, UAS-αSynA30P; UAS-lucRNAi

TH-Gal4, UAS-αSynA30P; UAS-Brm(wt)

TH-Gal4, UAS-αSynA30P; UAS-Brm RNAi

TH-Gal4, UAS-αSynA30P; UAS-Brm^DN^

Figure 4b:

TH-Gal4; UAS-dLrrk2^I1915T^ /+

TH-Gal4; UAS-dLrrk2^I1915T^/UAS-lucRNAi

TH-Gal4; UAS-dLrrk2^I1915T^/UAS-Brm(wt)

TH-Gal4; UAS- dLrrk2^I1915T^ / UAS-Brm RNAi

TH-Gal4, UAS-dLrrk2^I1915T^; UAS-Brm^DN^

Figure 4c:

TH-Gal4, UAS-Parkin RNAi /+

TH-Gal4, UAS-Parkin RNAi; UAS-lucRNAi

TH-Gal4, UAS-Parkin RNAi; UAS-Brm(wt)

TH-Gal4, UAS-Parkin RNAi; UAS-Brm RNAi

TH-Gal4, UAS- Parkin RNAi; UAS-Brm^DN^

Figure 4d:

*pink1*^B9^; TH-Gal4/+

*pink1*^B9^; TH-Gal4/UAS-lucRNAi

*pink1*^B9^; TH-Gal4/UAS-Brm(wt)

*pink1*^B9^; TH-Gal4/UAS-Brm RNAi

*pink1*^B9^; TH-Gal4/ UAS-Brm^DN^

Figure 4e:

TH-Gal4, UAS-αSynA30P/+

TH-Gal4, UAS-αSynA30P/UAS-dBVR RNAi

TH-Gal4, UAS-αSynA30P/UAS-dBVR (III)

TH-Gal4, UAS-αSynA30P/UAS-dBVR (II)

TH-Gal4, UAS-αSynA30P/UAS-dBVR (II); UAS-Brm^DN^

TH-Gal4, UAS-αSynA30P/UAS-dBVR (II); UAS-MEK RNAi

TH-Gal4, UAS-αSynA30P/UAS-dBVR (III); UAS-Aop^wt^

Figure 4f:

TH-Gal4; UAS- UAS-dLrrk2^I1915T^ /+

TH-Gal4; UAS-dLrrk2^I1915T^/UAS-dBVR RNAi

TH-Gal4; UAS-dLrrk2^I1915T^/UAS-dBVR(III)

TH-Gal4; UAS-dLrrk2^I1915T^ /UAS-dBVR(II)

TH-Gal4; UAS-dLrrk2^I1915T^ / UAS-dBVR (II); UAS-Brm^DN^

TH-Gal4; UAS-dLrrk2^I1915T^/UAS-dBVR(II); UAS-MEK RNAi

TH-Gal4; UAS-dLrrk2^I1915T^/UAS-dBVR(III); UAS-Aop^wt^

Figure 4g:

TH-Gal4, UAS-Parkin RNAi/+

TH-Gal4, UAS-Parkin RNAi/UAS-LucRNAi

TH-Gal4; UAS-Parkin RNAi/UAS-dBVR RNAi

TH-Gal4; UAS-Parkin RNAi /UAS-dBVR(III)

TH-Gal4; UAS-Parkin RNAi /UAS-dBVR(II)

TH-Gal4; UAS-Parkin RNAi / UAS-dBVR (II); UAS-Brm^DN^

TH-Gal4, UAS-Parkin RNAi/UAS-dBVR(II); UAS-MEK RNAi

TH-Gal4, UAS-Parkin RNAi/UAS-dBVR(III); UAS-Aop^wt^

Figure 4h:

*pink1*^B9^; TH-Gal4/+

*pink1*^B9^; TH-Gal4/UAS-lucRNAi

*pink1*^B9^; TH-Gal4/UAS-dBVR RNAi

*pink1*^B9^; TH-Gal4/UAS-dBVR(III)

*pink1*^B9^; TH-Gal4/UAS-dBVR(II)

*pink1*^B9^; TH-Gal4/UAS-dBVR(II); UAS-Brm^DN^

*pink1*^B9^; TH-Gal4/UAS-dBVR(II); UAS-MEK RNAi

*pink1*^B9^; TH-Gal4/UAS-dBVR(II); UAS-Aop^wt^

Figure 4i, j:

TH-Gal4, UAS-RFP; Pnt::EGFP

TH-Gal4, UAS-RFP; Pnt::EGFP/UAS-dBrm RNAi

Figure 4k, l:

TH-Gal4, UAS-RFP; Pnt::EGFP

TH-Gal4, UAS-RFP; Pnt::EGFP/UAS-dBrm (wt)

Figure 4m, n:

TH-Gal4, UAS-RFP; Pnt::EGFP

TH-Gal4, UAS-RFP; Pnt::EGFP/UAS-dBvr RNAi

TH-Gal4, UAS-RFP; Pnt::EGFP/UAS-dBvr II

**Figure 5**

Figure 5a, b:

*elav*-Gal4/+

*elav*-Gal4/UAS-lucRNAi

*elav*-Gal4/UAS-αSynA30P

*elav*-Gal4/UAS-dLrrk2^I1915T^

*elav*-Gal4/UAS-dPINK1 RNAi

*elav*-Gal4; UAS-dParkin RNAi

Figure 5c-e:

TH-Gal4; UAS-RFP/Pnt::EGFP

TH-Gal4, UAS-αSynA30P; UAS-RFP/Pnt::EGFP

TH-Gal4, UAS-RFP; UAS-dLrrk2^I1915T^/Pnt::EGFP

*pink1*^B9^; TH-Gal4, UAS-RFP; Pnt::EGFP

TH-Gal4, UAS-Parkin RNAi; UAS-RFP/Pnt::EGFP

Figure 5f:

TH-Gal4, UAS-αSynA30P/+

TH-Gal4, UAS-αSynA30P; UAS-lucRNAi

TH-Gal4, UAS-αSynA30P; UAS-Erk RNAi

TH-Gal4, UAS-αSynA30P; UAS-MEK RNAi

Figure 5g:

TH-Gal4; UAS- UAS-dLrrk2^I1915T^ /+

TH-Gal4; UAS- UAS-dLrrk2^I1915T^/UAS-lucRNAi

TH-Gal4; UAS- UAS-dLrrk2^I1915T^/UAS-Erk RNAi

TH-Gal4; UAS- UAS-dLrrk2^I1915T^/ UAS-MEK RNAi

Figure 5h:

TH-Gal4, UAS-Parkin RNAi /+

TH-Gal4, UAS-Parkin RNAi; UAS-lucRNAi

TH-Gal4, UAS-Parkin RNAi; UAS-Erk RNAi

TH-Gal4, UAS-Parkin RNAi; UAS-MEK RNAi

Figure 5i:

*pink1*^B9^; TH-Gal4/+

*pink1*^B9^; TH-Gal4/UAS-lucRNAi

*pink1*^B9^; TH-Gal4/UAS-Erk RNAi

*pink1*^B9^; TH-Gal4/UAS-MEK RNAi

Figure 5j:

TH-Gal4, UAS-αSynA30P/+

TH-Gal4, UAS-αSynA30P/UAS-LucRNAi

TH-Gal4, UAS-αSynA30P/UAS-Pnt RNAi

TH-Gal4, UAS-αSynA30P/UAS-Aop RNAi

TH-Gal4, UAS-αSynA30P/UAS-Aop^wt^

Figure 5k:

TH-Gal4; UAS-dLrrk2^I1915T^ /+

TH-Gal4; UAS-dLrrk2^I1915T^ /LucRNAi

TH-Gal4; UAS-dLrrk2^I1915T^/UAS-Pnt RNAi

TH-Gal4; UAS-dLrrk2^I1915T^/UAS-Aop RNAi

TH-Gal4; UAS-dLrrk2^I1915T^ /UAS-Aop^wt^

Figure 5l:

TH-Gal4, UAS-Parkin RNAi/+

TH-Gal4, UAS-Parkin RNAi/UAS-LucRNAi

TH-Gal4; UAS-Parkin RNAi/UAS-Pnt RNAi

TH-Gal4; UAS-Parkin RNAi /UAS-Aop RNAi

TH-Gal4, UAS-Parkin RNAi/UAS-Aop^wt^

Figure 5m:

*pink1*^B9^; TH-Gal4/+

*pink1*^B9^; TH-Gal4/UAS-lucRNAi

*pink1*^B9^; TH-Gal4/UAS-Pnt RNAi

*pink1*^B9^; TH-Gal4/UAS-Aop RNAi

*pink1*^B9^; TH-Gal4/UAS-Aop^wt^

Figure 5n-p:

TH-Gal4/+

TH-Gal4; UAS-αSynA30P

TH-Gal4; UAS-dLrrk2^I1915T^

TH-Gal4; UAS-dParkin RNAi

*pink1*^B9^; TH-Gal4/+

**Figure S2**

elav-Gal4/+

elav-Gal4/+; UAS-mitodsRed RNAi

elav-Gal4/+; UAS-dBVR II

elav-Gal4/+; UAS-BVRIII

elav-Gal4+; UAS-dBVR RNAi

**Figure S5**

TH-Gal4/+

TH-Gal4; UAS-αSynA30P

TH-Gal4; UAS-dLrrk2^I1915T^

TH-Gal4; UAS-dParkin RNAi

TH-Gal4; UAS-dPINK1 RNAi

*pink1*^B9^; TH-Gal4/+

**Figure S6**

TH-Gal4; UAS-RFP/Tub-mito-roGFP2

TH-Gal4, UAS-αSynA30P; UAS-RFP/ Tub-mito-roGFP2

TH-Gal4, UAS-RFP; UAS-dLrrk2^I1915T^/ Tub-mito-roGFP2

TH-Gal4, UAS-PINK1 RNAi; UAS-RFP/ Tub-mito-roGFP2

TH-Gal4, UAS-Parkin RNAi; UAS-RFP/ Tub-mito-roGFP2

**Figure S7**

TH-Gal4; UAS-RFP/UAS-roGFP2

TH-Gal4, UAS-αSynA30P; UAS-RFP/ UAS-roGFP2

TH-Gal4, UAS-RFP; UAS-dLrrk2^I1915T^/ UAS-roGFP2

TH-Gal4, UAS-PINK1 RNAi; UAS-RFP/ UAS-roGFP2

TH-Gal4, UAS-Parkin RNAi; UAS-RFP/ UAS-roGFP2

**Figure S8**

TH-Gal4; UAS-RFP/GstD-GFP

TH-Gal4, UAS-αSynA30P; UAS-RFP/GstD-GFP

TH-Gal4, UAS-RFP; UAS-dLrrk2^I1915T^/GstD-GFP

TH-Gal4, UAS-PINK1 RNAi; UAS-RFP/GstD-GFP

TH-Gal4, UAS-Parkin RNAi; UAS-RFP/GstD-GFP
